# Association between long-term ambient air pollution exposure and cognitive disorders among older adults in China: the mediating role of lifestyle

**DOI:** 10.7189/jogh.16.04307

**Published:** 2026-10-19

**Authors:** Yu Zhang, Jiaqi Yang, Yixin Zhang, Quan Yuan, Yanan Li, Ruizheng Ding, Guanling Song, Qiang Niu, Yunhua Hu, Yizhong Yan

**Affiliations:** 1 Department of Preventive Medicine, School of Public Health, Shihezi University, Shihezi, Xinjiang, China; 2 University of Melbourne, Melbourne, Victoria, Australia; 3 Key Laboratory for Prevention and Control of Crucial Emerging Infectious Diseases and Public Health Security of The Xinjiang Production and Construction Corps, Shihezi, Xinjiang, China; 4 Key Laboratory of Preventive Medicine, Shihezi University, Shihezi, Xinjiang, China; 5 Key Laboratory of Xinjiang Endemic and Ethnic Diseases (Ministry of Education), School of Medicine, Shihezi University, Shihezi, Xinjiang, China

**Keywords:** cognitive disorders, ambient air pollution, lifestyle, Bayesian weighted quantile and regression, mediation analysis

## Abstract

**Background:**

Cognitive disorders (CDs) are a major public health concern in ageing societies. The association between air pollution and CDs has received considerable attention; however, the moderating role of lifestyle remains unclear. We aimed to explore the complex associations among lifestyle, its dynamic changes, ambient air pollution exposure, and CDs in older adults.

**Methods:**

We included data from the Chinese Longitudinal Healthy Longevity Study. We used Cox proportional hazards models, Bayesian weighted quantile sum models, and mediation analysis to explore associations between lifestyle, air pollution, and CDs risk.

**Results:**

Per 10 μg/m^3^ increase in exposure to particulate matter with diameters ≤2.5 μm (hazard ratio (HR) = 1.005; 95% confidence interval (CI) = 1.001–1.009), sulphates (HR = 1.030; 95% CI = 1.003–1.056), ozone (HR = 1.006; 95% CI = 1.001–1.011), nitrates (HR = 1.020; 95% CI = 1.003–1.037), and ammonium (HR = 1.033; 95% CI = 1.005–1.062) was associated with an increased risk of CDs. The mixture exposure showed a significant positive association with CDs (odds ratio = 1.028; 95% CI = 1.006–1.040). Adherence to both intermediate (HR = 0.732; 95%CI = 0.569–0.940) and favourable (HR = 0.488; 95%CI = 0.336–0.710) lifestyles was associated with a reduced risk of CDs compared to an unfavourable lifestyle, and the protective effect was more significant at high pollution exposure levels (about 52% lower risk). Lifestyle mediated the effect by 8.4–9.4% in the association between single pollutants and CDs, and 9.1–9.5% in the association between mixed pollutants and CDs. Ozone had the highest weight in the pollution mixture.

**Conclusions:**

Air pollution increased the risk of CDs among older adults, which was partially offset by a healthy lifestyle. Lifestyle interventions may help address some challenges inherent in controlling air pollution in ageing societies.

Cognitive disorders (CDs) are diseases that seriously affect individuals’ cognitive functions, such as memory, judgment, attention, and language [1], and have become one of the main causes of disability and mortality among elderly populations [2]. They can be subdivided into mild cognitive impairment and dementia, where patients with dementia exhibit more pronounced cognitive decline, and their quality of life is significantly reduced [3]. According to statistics from 2019, the number of patients with dementia worldwide has reached 57 million, with China being one of the most severely affected countries, accounting for 27% of dementia cases [4]. According to the National Bureau of Statistics, the number of older adults aged ≥65 years in China reached 176 million in 2019, and is projected to rise to 240 million by 2030 and 365 million by 2050 [5]. As the global population progressively ages, the prevalence of cognitive impairment is projected to continue rising, which not only poses serious challenges to individuals and families, but also puts enormous pressure on public health systems [6].

Lifestyle, an important factor affecting individual health, has received increasing attention in research investigating CDs in recent years. A healthy lifestyle, including healthy living habits, adequate rest, and positive social interactions, has a positive effect on maintaining brain health and delaying cognitive decline, whereas an unfavourable lifestyle, such as smoking, excessive drinking, and lack of leisure and exercise, may increase the risk of developing CDs [7]. Therefore, improving lifestyle to prevent and manage CDs has become a trending research topic and a practical direction.

However, when exploring the impact of lifestyle on CDs, environmental factors must not be overlooked. Air pollution is a growing global problem, and multiple studies have confirmed its negative effects on brain function. Long-term exposure to high concentrations of air pollutants, such as particulate matter with diameters ≤2.5 μm (PM_2.5_), sulphates (SO_4_), ozone (O_3_), nitrates (NO_3_), and ammonium (NH_4_), may damage brain structure and function, and further accelerate cognitive decline [8]. For example, a prospective cohort study from the USA found that, in New York, adults aged ≥65 years in areas with higher PM_2.5_ experienced a faster rate of cognitive decline [9].

Although numerous studies have investigated the association between lifestyle and environmental factors and the risk for CDs, significant knowledge gaps persist, especially among the elderly population. First, although lifestyle factors are interrelated, few studies have developed integrated lifestyle indices to reflect and assess their impacts on the adverse effects of ambient air pollution exposure. These studies focused only on baseline lifestyle and did not account for changes during follow-up. Second, solid evidence is lacking on the extent to which adherence to a healthy lifestyle modifies the association between ambient air pollution exposure and the incidence of CDs. Additionally, it is unclear whether these effects are consistent across healthy lifestyle levels, types of ambient air pollutants, and exposure levels. More importantly, in highly polluted environments, how individuals can adopt effective protective measures to reduce CD risk remains a critical issue that warrants further investigation. Accordingly, we aimed to explore the complex association between lifestyle and its dynamic changes, exposure to ambient air pollution, and CDs among older adults based on data from a prospective cohort study investigating the health of the elderly in China, and to provide scientific evidence for finding effective strategies and interventions to balance economic development and physical health.

## METHODS

### Study population

We collected the data from the Chinese Longitudinal Healthy Longevity Study (CLHLS), a comprehensive prospective national cohort study [10]. The CLHLS is one of the largest nationwide health surveys in China, covering 866 counties and cities in 23 regions (18 provinces, 1 autonomous region, and 4 municipalities directly under the Central Government) and accounting for approximately 85% of the country's total population. Since its inception in 1998, the CLHLS has conducted eight consecutive waves (1998, 2000, 2002, 2005, 2008–2009, 2011–2012, 2014, and 2018–2019) (Figure S1 in the **Online Supplementary Document**).

The CLHLS collected in-person interview data on socioeconomic characteristics, lifestyle, health status, cognitive function, and other factors among older adults aged ≥65 years and investigated potential risk factors affecting longevity in China. All respondents signed a written informed consent form before participation [11].

We used three longitudinal waves of CLHLS data: 2011–2012, 2014, and 2018–2019, covering 2011–2019. We designed this study as a fixed cohort, and we included the entire study population at baseline (2011–2012) and followed them up in 2014 and 2018–2019 to collect additional information on lifestyle and outcomes. We included participants aged ≥65 years with no history of dementia, stroke, Parkinson disease, epilepsy, or CDs at baseline. Following the inclusion criteria, we excluded those who were aged <65 years (n = 75), individuals with dementia, stroke, Parkinson disease, or epilepsy at baseline (n = 370), and those with CDs (Mini-Mental State Examination (MMSE) score <18) at baseline (n = 2039). Additionally, 4509 participants were deceased, and 503 were lost to follow-up. Ultimately, the final sample included data from 2269 participants (Figure S2 in the **Online Supplementary Document**).

### Exposure to ambient air pollution

We obtained data on ambient PM_2.5_, SO_4_, O_3_, NO_3_, and NH_4_ from the Tracking Air Pollution in China [12], which provides gridded estimates at 0.1° × 0.1° spatial resolution. The PM_2.5_ concentrations were estimated using a two-stage machine learning model that integrated multisource data, including ground-based observations, satellite-derived aerosol optical depth, chemical transport model simulations, and ancillary meteorological and land-use variables. A tree-based gap-filling method was applied to address these data gaps [13,14].

We derived the chemical composition of PM_2.5_ (*e.g.* SO_4_, NO_3_, NH_4_) from Community Multiscale Air Quality (CMAQ) model simulations and corrected it to reduce biases. The CMAQ dust emission module was improved to better represent dust contributions from windblown mineral dust originating from deserts such as the Taklamakan and Gobi Deserts. In the CMAQ dust emission module, these contributions are represented by source-dependent speciation profiles to reduce simulation bias for trace metals [14,15]. We used an eXtreme Gradient Boosting algorithm to adjust simulated component concentrations by incorporating ground-based PM_2._5 observations. This approach refined the relative contributions of individual components to the total PM_2.5_, yielding more accurate estimates of SO_4_, NO_3_, NH_4_, and other pollutants [13,14].

For O_3_, Tracking Air Pollution established two random forest models: one to correlate O_3_ observations, CMAQ-simulated O_3_ concentrations, and other variables, and another to incorporate satellite remote-sensing O_3_ vertical profiles based on the previous model. Because satellite remote sensing data were unavailable, the second model's predictions showed spatiotemporal discontinuities. Second, we established an elastic network regression model to integrate the predictions of the two random forest models, which improved accuracy and achieved complete spatiotemporal coverage. Finally, we used the spatiotemporal variance-covariance function to model correlations in the residual errors. On this basis, the spatiotemporal Kriging interpolation was used to further integrate site-based observed signals into the final predicted value [16].

To characterise long-term air pollution exposure, we obtained annual data for the five pollutants from 2010 to 2018. We used the autoregressive integrated moving average (ARIMA) model to fit annual concentration trends for each region. The inclusion of 2010 data (one year prior to the study baseline) helped to capture the pre-baseline exposure background and provided a more stable time-series for the ARIMA model estimation [17]. We calculated the average of the fitted values from 2010 to 2018 as the cumulative exposure level for each participant. Subsequently, we assigned air pollution exposure to each participant based on the regions of residence obtained through the baseline questionnaire, without conducting biological or personal exposure measurements.

### Healthy lifestyles

We used baseline lifestyle information to analyse its association with the outcome of CDs. Additionally, we collected lifestyle information at the time of the outcome, and defined dynamic changes in lifestyle type from baseline to outcome as lifestyle transition. We also analysed the association between lifestyle transition and CDs [18]. Based on the World Health Organization’s ‘Self-care in Dementia’ and previous studies [7,10,19], we constructed a healthy lifestyle score to assess modifiable risk factors including smoking, drinking, residential situation, sleep quality, social activities, and leisure activities. Trained and tested investigators collected lifestyle information in the CLHLS through questionnaires [20]. Smoking status at present was derived from the question ‘Smoking or not at present?’ (yes/no), and a similar question format assessed drinking status. Participants’ residential situation was derived from the question ‘Co-residence’(with household member (s)/alone/in an institution). We merged ‘with household member (s)’ and ‘in an institution’ into ‘Living with family’. Participants’ sleep quality was derived from the question ‘How about the quality of your sleep?’ (very good/good/so so/bad/very bad). We merged ‘very good’ and ‘good’ into ‘good sleep quality’ and the rest into ‘bad sleep quality’. Social activities were assessed with the question ‘Do you take part in some social activities at present?’ (almost every day/not daily, but once a week/not weekly, but at least once a month/not monthly, but sometimes/never). We considered participation in social activities at least once a week to be healthy. Similar questions and responses were used for leisure activities (*i.e.* doing housework, growing vegetables, performing garden housework, reading books or newspapers, keeping pets, playing cards, and watching TV). Each of these six factors was assigned a score of 0–1 (Table S1 in the **Online Supplementary Document**), and the total number of modifiable lifestyle factors was calculated on a scale of 0–6, with higher scores indicating greater adherence to healthy lifestyles. We divided participants into three lifestyle categories based on their scores: unfavourable (0–1 point), intermediate (2–4 points), and favourable (5–6 points) [7].

### CDs

The CLHLS assessed the cognitive function of participants using the Chinese version of the MMSE, which has demonstrated high validity and reliability in the elderly Chinese population [21–23]. The CLHLS personnel conducted the MMSE survey after undergoing unified training. To improve survey techniques and ensure survey quality, each region provided thorough training for surveyors and produced training videos. Through questionnaire explanation, skill introduction, simulated surveys, discussion, and summary, the investigator clarified the survey purpose, understood the survey plan, mastered survey skills, paid attention to the overall survey structure, and completed the survey task as required within the unified time. In summary, during the investigation process, all investigators received the same training and assessment to standardise their capabilities. The elderly needed to answer all questions related to cognitive functions themselves; no one else could answer on their behalf. If the elderly had difficulty answering the questions, investigators had to find out why they could not answer.

The MMSE comprises five domains: orientation to time and place; registration, attention and calculation; recall; and language. The MMSE scores range from 0 to 30, with higher scores indicating better cognitive function. Of the participants in the CLHLS, 81.16% completed only primary or lower education level and thereby may have a low MMSE score at baseline. Thus, we identified participants with CDs based on an MMSE score of <18 and a decrease of >4 points from baseline, consistent with previous studies [8,24–26].

### Covariates

The CLHLS obtained the data for age, sex (male/female), marital status (married/single: including separated, divorced, and never married), and residence (rural/urban) from standard questionnaires [27]. Self-reported diseases included hypertension, diabetes, heart disease, and chronic lung disease.

### Statistical analyses

We stratified the participants into three lifestyle groups (*i.e.* unfavourable, intermediate, and favourable). We performed trend analyses using linear regression for continuous variables, and Wald χ^2^ tests for categorical variables across these groups, with trend *P*-values calculated for each lifestyle category. We developed a directed acyclic graph to guide covariate selection (Figure S3 in the **Online Supplementary Document**).

We also stratified participants by air pollutant exposure into quintile 1 (Q1), quintile 2 (Q2), quintile 3 (Q3), quintile 4 (Q4), and quintile 5 (Q5) to assess the gradient effect of exposure. At the same time, we combined Q2 to Q5 for a binary classification to directly observe differences between low and high exposure. We also performed additional stratified analyses to examine the effects of lifestyle and CDs within different air pollutant exposure subgroups (*i.e.* Q1, Q2, Q3, Q4, Q5, and Q2–Q5).

In the Cox regression models, we used follow-up time (years) as the time metric. Participants were followed up from the baseline survey (2011–2012), with follow-up ending at the CD evaluation at the end of follow-up (2018–2019). We did not apply censoring or staggered entry in the Cox regression model. We used Cox regression models to examine associations between air pollutant exposure (per 10 g/m^3^ increase) and CDs, as well as between lifestyle and CDs, expressed as hazard ratios (HRs) and corresponding 95% confidence intervals (CIs). We also used Cox regression models to analyse the association between lifestyle changes from baseline to outcome and the risk of CDs. The analysis also considered the effects of different air pollutant exposure subgroups. We observed no evidence of violating the proportional-hazards assumption (tested by Schoenfeld’s residuals) in the Cox regression models.

We further divided participants into two lifestyle categories, including unfavourable (0–1 points) and intermediate and favourable (2–6 points), to test for potential additive interactions. We selected potential confounders that were significant in baseline comparisons or clinically relevant for multivariate adjustment models. We adjusted covariates using two main models: an unadjusted model (model 1), and a model adjusted for age, sex, marital status, place of residence, hypertension, diabetes, heart disease, and lung disease (model 2).

We used a new Bayesian weighted quantile regression, known as Bayesian Weighted Quantile Sum (BWQS), to assess the effects of mixed air pollution exposure on CDs [28,29]. In the BWQS, the estimated coefficients for mixed air exposure determined the association between overall air pollutants and CDs, whereas the estimated coefficients mapped to weights represented the relative contributions of the individual components to the mixture. The BWQS regression retained many features of the frequency-theoretic weighted quantile sum (WQS) regression, but did not require *a priori* specification of the effects for a single direction on the entire mixing or splitting of the original data, thus improving the statistical power, flexibility, and stability of the estimation [29].

The BWQS index represented the mixed exposure levels of the five air pollutants and served as the exposure metric in the mediation analyses. The BWQS index was composed of the weighted sum of the concentrations of each air pollutant [30].

### Sensitivity analysis

We performed a series of sensitivity analyses to assess the robustness of the findings. We applied WQS regression to assess the effects of mixed air pollution exposure on CDs because it performs well in characterising environmental mixtures. We used the ‘gWQS’ package in *R* to calculate the WQS index as the weighted sum of each air pollutant concentration. Specifically, we entered the five ambient air pollutants (*i.e.* PM_2.5_, SO_4_, O_3_, NO_3_, and NH_4_) into the model. In practice, we first scored individual pollutant concentrations into quartiles. To evaluate the mixture effect, we randomly partitioned the data set into a training data set (40%) for weight estimation and a validation data set (60%) for hypothesis testing (validation = 0.6). We ran a bootstrapping procedure with 1000 iterations to ensure stable weight estimates. Both positive and negative directionality constraints were applied to evaluate potential bidirectional associations with CDs. In the mediation analysis of mixed air pollutant exposure, the WQS index was constructed by adding the product of each pollutant's concentration and its corresponding estimated weight from the WQS model to represent mixed air pollution exposure as the exposure metric in the mediation analyses [30]. Concurrently, we used quantile g-computation (Qgcomp) regression to jointly assess the effects of mixed air pollutants on CDs and determine their complex associations with lifestyle [31].

We used *R*, version 4.4.2. (R Core Team, Vienna, Austria) for all analyses.

## RESULTS

### Baseline characteristics

A total of 2269 participants were included. The mean (x̄) age was 77.86 (standard deviation (SD) = 8.40) years, and 48.6% were male (**Table 1**).

**Table 1:** Baseline characteristics of participants by lifestyle categories*

		Lifestyle category			
	**Total (n = 2269)**	**Unfavourable (n = 340)**	**Intermediate (n = 1670)**	**Favourable (n = 259)**	***P*-value†**
**Age in years, x̄ (SD)**	77.86 (8.40)	76.89 (8.14)	77.82 (8.42)	79.35 (8.42)	0.003
**Sex**					<0.001
Male	1,103 (48.6)	276 (81.2)	739 (44.3)	88 (34.0)	
Famale	1,166 (51.4)	64 (18.8)	931 (55.7)	171 (66.0)	
**Marital status**					<0.001
Single	1,018 (44.9)	107 (31.5)	713 (42.7)	198 (76.4)	
Married	1,251 (55.1)	233 (68.5)	957 (57.3)	61 (23.6)	
**Residence**					0.068
Rural	2,112 (93.1)	322 (94.7)	1,557 (93.2)	233 (90.0)	
Urban	157 (6.9)	18 (5.3)	113 (6.8)	26 (10.0)	
**Hypertension**	688 (30.3)	80 (23.5)	508 (30.4)	100 (38.6)	<0.001
**Diabetes**	83 (3.7)	7 (2.1)	68 (4.1)	8 (3.1)	0.172
**Heart disease**	240 (10.6)	29 (8.5)	180 (10.8)	31 (12.0)	0.348
**Chronic lung disease**	228 (10.0)	31 (9.1)	176 (10.5)	21 (8.1)	0.397
**Residential situation**					<0.001
Living alone	1,797 (79.2)	326 (95.9)	1,417 (84.9)	54 (20.8)	
Living with family	472 (20.8)	14 (4.1)	253 (15.1)	205 (79.2)	
**Smoking**	1,779 (78.4)	76 (22.4)	1,446 (86.6)	257 (99.2)	<0.001
**Drinking**	1,770 (78.0)	93 (27.4)	1,422 (85.1)	255 (98.5)	<0.001
**Sleep quality**					<0.001
Bad sleep quality	827 (36.4)	198 (58.2)	615 (36.8)	14 (5.4)	
Good sleep quality	1,442 (63.6)	142 (41.8)	1,055 (63.2)	245 (94.6)	
**Leisure activities**					<0.001
Do housework more than once a week	1,731 (76.3)	205 (60.3)	1,307 (78.3)	219 (84.6)	
Grow vegetables more than once a week	1,497 (66.0)	210 (61.8)	1,096 (65.6)	191 (73.7)	
Garden housework more than once a week	547 (24.1)	85 (25.0)	394 (23.6)	68 (26.3)	
Read more than once a week	423 (18.6)	79 (23.2)	290 (17.4)	54 (20.8)	
Keep pets more than once a week	779 (34.3)	128 (37.6)	580 (34.7)	71 (27.4)	
Play cards more than once a week	348 (15.3)	71 (20.9)	234 (14.0)	43 (16.6)	
Watch TV more than once a week	1,816 (80.0)	271 (79.7)	1,340 (80.2)	205 (79.2)	
**Social activities more than once a week**	164 (7.2)	1 (0.3)	77 (4.6)	86 (33.2)	<0.001

SD – standard deviation, x̄ – mean

*Values are presented as n (%) unless specified otherwise.

†*P*-values were derived from analysis of variance for age, and from independent-samples *t*-test and K independent-samples tests of categorical variables for categorical variables.

In the low-level air pollution exposure group (Q1), average exposure levels (in μg/m^3^) were observed for PM_2.5_ (x̄ = 27.48; SD = 5.46), SO_4_ (x̄ = 5.44; SD = 0.96), O_3_ (x̄ = 99.10; SD = 2.04), NO_3_ (x̄ = 3.93; SD = 1.30), NH_4_ (x̄ = 4.23; SD = 4.82). In the high-level air pollution exposure group (Q2–Q5), the x̄ exposure levels (in μg/m^3^) were 56.88 (SD = 18.05) for PM_2.5_, 10.61 (SD = 3.05) for SO_4_, 124.46 (SD = 15.25) for O_3_, 11.99 (SD = 4.91) for NO_3_, and 9.14 (SD = 2.63) for NH_4_ (Table S2 in the **Online Supplementary Document**).

We also calculated correlations between various lifestyle factors (Figure S4 in the **Online Supplementary Document**). The highest correlation was observed between ‘non-smoking’ and ‘non-drinking’ (*r* = 0.31).

We divided participants into lifestyle groups: unfavourable (n = 340; 15.0%), intermediate (n = 1670; 73.6%), and favourable (n = 259; 11.4%). Adults with a favourable lifestyle were more likely to be female, single, live with family, have good sleep quality, and engage in frequent social activities. Smoking and alcohol consumption were more prevalent among adults with unfavourable lifestyles. Hypertension prevalence was significantly higher in the favourable (38.6%) compared to the unfavourable (23.5%) lifestyle group (**Table 1**).

The three lifestyle groups exhibited the greatest divergence in cohabitation status and the least in leisure activities. In the unfavourable lifestyle group, most older adults (87.1%) had two desirable lifestyle factors, with leisure activities being the most prevalent. In the intermediate lifestyle group, 52.6% of older adults had four ideal lifestyle factors, and nearly 90% abstained from smoking, alcohol consumption, and leisure activities. In the favourable lifestyle group, nearly 80% of older adults were non-smokers, alcohol-free, had good sleep hygiene, lived with family, and engaged in regular leisure activities. Moreover, 33% engaged in social activities, a proportion significantly higher than that in the other two groups (Figure S5 in the **Online Supplementary Document**).

The exposure concentrations of ambient air pollutants were comparable between individuals with unfavourable and favourable lifestyles; however, both groups had marginally higher levels than the intermediate lifestyle group (Table S3 in the **Online Supplementary Document**).

### Association between air pollution exposure and lifestyle with the incidence of CDs

During the seven-year follow-up period, 536 participants had CDs. After adjusting for covariates, each 10 μg/m^3^ increase in exposure was associated with an elevated risk for CDs: PM_2.5_ (HR = 1.005; 95% CI = 1.001–1.009), SO_4_ (HR = 1.030; 95% CI = 1.003–1.056), O_3_ (HR = 1.006; 95% CI = 1.001–1.011), NO_3_ (HR = 1.020; 95% CI = 1.003–1.037), and NH_4_ (HR = 1.033; 95% CI = 1.005–1.062) (**Table 2**). Conversely, adherence to both intermediate (HR = 0.732; 95% CI = 0.569–0.940) and favourable (HR = 0.488; 95% CI = 0.336–0.710) lifestyles was associated with a reduced risk of CDs (Table S4 in the **Online Supplementary Document**).

**Table 2:** Associations between air pollution (per 10 μg/m^3^ increase) and CDs with and without adjustment for lifestyle

	HR (95% CI)	
	**Unadjusted for lifestyle**	Adjusted for lifestyle*
**PM_2.5_**	1.001 (0.997–1.006)	1.005 (1.001–1.009)
**SO_4_**	1.007 (0.981–1.033)	1.030 (1.003–1.056)
**O_3_**	1.002 (0.997–1.008)	1.006 (1.001–1.011)
**NO_3_**	1.005 (0.989–1.022)	1.020 (1.003–1.037)
**NH_4_**	1.007 (0.980–1.036)	1.033 (1.005–1.062)

CD – cognitive disorders, CI – confidence interval, HR – hazard ratio, NH_4_ – compounds containing ammonium ions, NO_3_ – compounds containing nitrate ions, O_3_ – ozone, PM_2.5_ – particulate matter with an aerodynamic diameter ≤2.5 mm, SO_4_ – compounds containing sulphate ions

*Model was adjusted for age, sex, marital status, residence, hypertension, diabetes, heart disease, and chronic lung disease.

### Association between lifestyle and its dynamic changes and CDs under different concentrations of air pollution

When stratifying by specific air pollutant exposure levels, the favourable lifestyle group consistently showed a lower risk of cognitive impairment than the unfavourable lifestyle group in Q1, Q2, Q3, and Q5. At high levels of air pollution exposure (Q2–Q5), a favourable lifestyle was protective against CDs compared to an unfavourable lifestyle (**Figure 1**). The risk of developing CDs was reduced by 29% in the intermediate and by 52% in the favourable lifestyle group. At low levels of air pollution exposure, adherence to a favourable lifestyle minimised the risk of CDs (Table S5 in the **Online Supplementary Document**).

**Figure 1:**
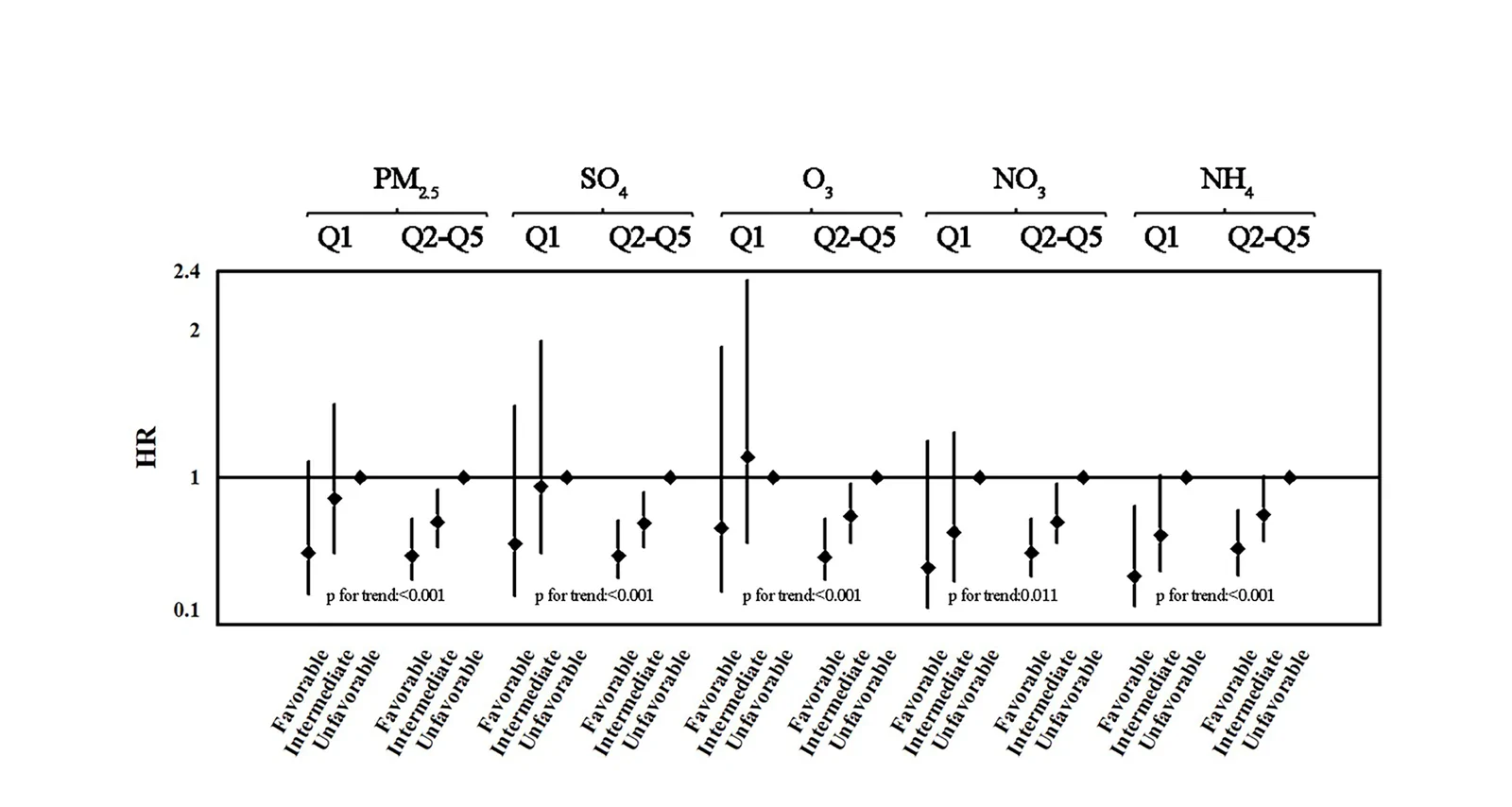
Joint effects of lifestyle and air pollutant exposure on the incidence of CDs. Unfavourable lifestyle as the reference; the model was adjusted for age, sex, marital status, residence, hypertension, diabetes, heart disease, and chronic lung disease. CD – cognitive disorders, CI – confidence interval, HR – hazard ratio, NH_4_ – compounds containing ammonium ions, NO_3_ – compounds containing nitrate ions, O_3_ – ozone, PM_2.5_ – particulate matter with an aerodynamic diameter ≤2.5 mm, SO_4_ – compounds containing sulphate ions.

At high air pollution exposure levels (Q2–Q5), individual lifestyle factors significantly influenced the risk for CDs, and living with family, social activities, and leisure activities exhibited a significant protective effect. As lifestyle scores increased (from 0 to 6), the risk for CDs gradually decreased, especially at high air pollution exposure levels (Q2–Q5), where the risk was substantially reduced (Table S6 in the **Online Supplementary Document**).

Notably, among those with an intermediate baseline lifestyle, 6.13% reverted to an unfavourable one, 48.74% stayed intermediate, and 18.73% transitioned to a favourable lifestyle (Table S7 in the **Online Supplementary Document**). After adjusting for covariates, sticking to or shifting to an intermediate or favourable lifestyle was notably associated with a lower risk of CDs across different air pollutants (**Table 3**; Table S8–11 in the **Online Supplementary Document**). For PM_2.5_ exposure, among participants with an intermediate lifestyle at baseline, adherence to or transition to a favourable lifestyle was significantly associated with a lower risk of CDs, particularly at high exposure levels (Q2–Q5) (HR = 0.27; 95% CI = 0.18–0.41, *P* = 0.009).

**Table 3:** Associations between lifestyle dynamic changes and the risk of CDs under exposure to PM_2.5_

		Q1*		Q2–Q5*	
**Outcome by baseline lifestyle**	**Events/n**	**HR (95%CI)**	***P*-value**	**HR (95%CI)**	***P*-value**
Unfavourable lifestyle					
*Unfavourable lifestyle*	19/73	ref		ref	
*Intermediate lifestyle*	53/230	1.92 (0.57–6.49)	0.297	0.96 (0.53–1.74)	0.887
*Favourable lifestyle*	8/37	2.26 (0.49–10.52)	0.299	0.78 (0.25–2.43)	0.674
Intermediate lifestyle					
*Unfavourable lifestyle*	67/139	ref		ref	
*Intermediate lifestyle*	252/1,106	0.39 (0.19–0.80)	0.010	0.40 (0.30–0.55)	<0.001
*Favourable lifestyle*	60/425	0.32 (0.14–0.75)	0.009	0.27 (0.18–0.41)	<0.001
Favourable lifestyle					
*Unfavourable lifestyle*	5/26	ref		ref	
*Intermediate lifestyle*	36/169	0.56 (0.10–3.17)	0.508	1.42 (0.42–4.88)	0.575
*Favourable lifestyle*	7/64		0.976	1.28 (0.31–5.25)	0.736

CD – cognitive disorder, CI – confidence interval, HR – hazard ratio, PM_2.5_ – particulate matter with an aerodynamic diameter ≤2.5 mm, ref – reference

*Adjusted for age, sex, marital status, residence, hypertension, diabetes, heart disease, and chronic lung disease.

### Association between air pollution exposure and CDs under different lifestyle categories and the mediating role of lifestyle

After adjusting for covariates, in the intermediate lifestyle group, for every 10 μg/m^3^ increase in PM_2.5_ (HR = 1.005; 95% CI = 1.001–1.011), SO_4_ (HR = 1.033; 95% CI = 1.002–1.064), O_3_ (HR = 1.006; 95% CI = 1.001–1.012), NO_3_ (HR = 1.023; 95% CI = 1.003–1.042), and NH_4_ (HR = 1.037; 95% CI = 1.005–1.071), the risk of CDs increased (Table S12 in the **Online Supplementary Document**). However, the association between PM_2.5_, SO_4_, O_3_, NO_3_, and NH_4_ with CDs was not significant in the unfavourable or favourable lifestyle groups. Lifestyle also mediated the effect of air pollution exposure on CDs. The proportions of mediation effects in the associations between air pollutants and CDs were the highest for O_3_ (9.42%), followed by SO_4_ (9.01%), NH_4_ (8.84%), NO_3_ (8.81%), and PM_2.5_ (8.42%) (Figure S6 in the **Online Supplementary Document**).

In the subgroup analysis by dichotomised lifestyle, for other air pollutants at high exposure levels (Q2–Q5), the intermediate and favourable lifestyle groups exhibited significant protection against CDs compared with the unfavourable lifestyle group (**Figure 2**). Except for O_3_, the reduction in CDs risk associated with a healthy lifestyle was significant in females at other high levels of air pollution exposure (*P* < 0.05) (Table S13 in the **Online Supplementary Document**).

**Figure 2:**
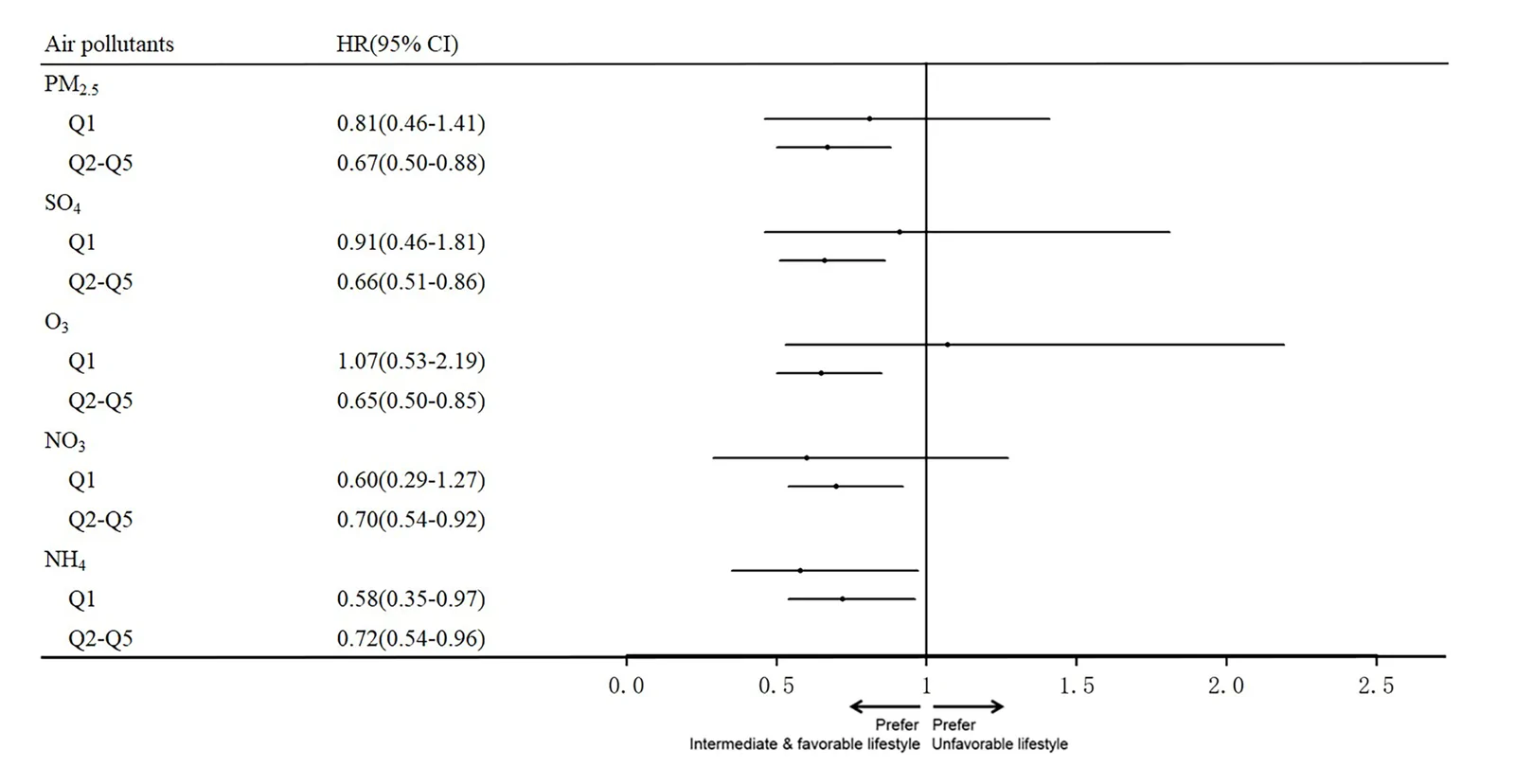
Additive interaction analysis of the effect of dichotomised lifestyle on the association between ambient air pollutant exposure and CDs. The model was adjusted for age, sex, marital status, residence, hypertension, diabetes, heart disease, and chronic lung disease. CD – cognitive disorders, CI – confidence interval, HR – hazard ratio, NH_4_ – compounds containing ammonium ions, NO_3_ – compounds containing nitrate ions, O_3_ – ozone, PM_2.5_ – particulate matter with an aerodynamic diameter ≤2.5 mm, SO_4_ – compounds containing sulphate ions.

### Association between exposure to mixed air pollutants (per 10 μg/m3) and CDs and the mediating role of lifestyle

The BWQS model revealed a significant association between CDs and each one-quartile increase in mixed exposure to the five air pollutants, with a coefficient of 0.027 (95% CI = 0.006–0.039), corresponding to an adjusted odds ratio (OR) of 1.028 (95% CI = 1.006–1.040) in all participants. Of the five pollutants, O_3_ (22.7%) had the highest weight in the BWQS model. In the mediation analysis for mixed air pollution exposure, lifestyle significantly mediated the association between mixed air pollution exposure and CDs, with a mediating effect proportion of 9.41% (*P* = 0.030) (**Figure 3**, Panels A–C).

**Figure 3:**
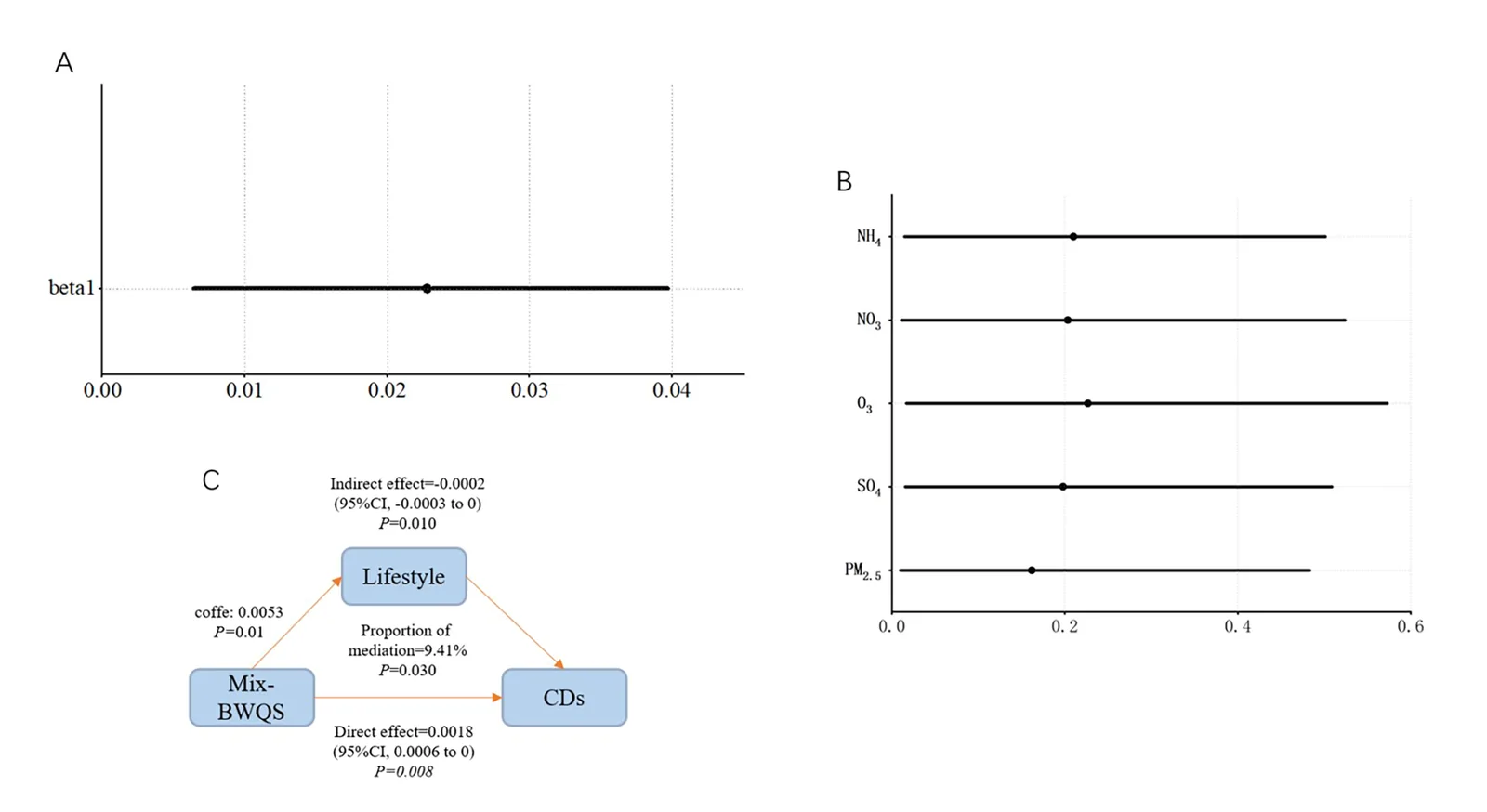
Association between exposure to air pollutants, CDs, and the mediating role of lifestyle. **Panel A.** Estimates (coefficients) of the association between Mix-BWQS and CDs in the BWQS model. **Panel B.** Weights with 95% credible intervals for each air pollutant exposure. **Panel C.** The mediation effect of lifestyle categories on Mix-BWQS in CDs. BWQS model was adjusted for age, sex, marital status, residence, hypertension, diabetes, heart disease, and chronic lung disease; Mix-BWQS is the sum of the products of the weights obtained from the BWQS model and concentrations of various air pollutants. BWQS – Bayesian Weighted Quantile Sum, CD – cognitive disorders, CI – confidence interval, NH_4_ – compounds containing ammonium ions, NO_3_ – compounds containing nitrate ions, O_3_ – ozone, PM_2.5_ – particulate matter with an aerodynamic diameter ≤2.5 mm, SO_4_ – compounds containing sulphate ions.

### Sensitivity analysis

In the WQS positive model, mixed air pollution was positively associated with the risk of CDs (OR = 1.151; 95% CI = 1.008–1.314). Of the five pollutants, O_3_ (32.6%) had the highest weight in the WQS model. The proportion of lifestyle-mediated effects on CDs risk was 9.07% (*P* = 0.012) (Table S14 and Figure S7, Panels A and C in the **Online Supplementary Document**). These results corroborate those of the BWQS model.

In the Qgcomp model, the risk for CDs increased significantly with each one-quartile increase in air pollution exposure (OR = 1.219; 95% CI = 1.089–1.365). Consistent with BWQS and WSQ results, O_3_ (54.9%) had the highest positive weight in the Qgcomp model (Table S14 and Figure S7, Panel D in the **Online Supplementary Document**). The two models further suggested a strong association between increased air pollution exposure and a significantly elevated risk for CDs, with lifestyle exerting a significant mediating effect.

## DISCUSSION

We explored the complex association between lifestyle, ambient air pollution exposure, and CD risk among older adults through an in-depth analysis of longitudinal data from the CLHLS. The results showed that exposure to ambient air pollution, particularly higher concentrations of PM_2.5_, SO_4_, O_3_, NO_3_, and NH_4_, was positively associated with CD risk. Simultaneously, adhering to a healthy lifestyle, including no smoking, no drinking, good sleep quality, and active social activities and leisure activities (*e.g.* doing housework, growing vegetables, gardening activities, reading newspapers or watching television, keeping pets, playing cards), had a significant protective role in reducing the risk of CDs caused by air pollution. This is especially important for reducing CD risk among older adults facing uncontrolled air pollution.

We confirmed an association between ambient air pollution exposure and CD risk. These results align with findings from multiple studies worldwide, indicating that air pollution is an important modifiable risk factor for cognitive decline and dementia [32,33]. Notably, O_3_ had the highest weight in both the BWQS and WQS models, suggesting that O_3_ may have a particularly important effect on cognitive health. Although O_3_ is generally believed to primarily affect the respiratory system, emerging evidence indicates that it may also affect the nervous system through oxidative stress and inflammatory responses [24,34]. Therefore, reducing O_3_ levels should be prioritised when formulating air pollution prevention and control strategies.

Our results highlight the importance of a healthy lifestyle in preventing CDs in older adults. In recent years, modifiable risk factors have received increasing attention and are gradually emerging as key individual-level correlates. Numerous clinical trials and observational studies have explored ways to reduce the harmful effects of air pollution on CDs at the individual level; however, their conclusions remain limited [35,36]. First, studies that estimated the impact of a single lifestyle factor on CDs often failed to reflect the overall lifestyle [37]. These studies focused only on baseline lifestyle and did not account for changes during follow-up. In addition, the mediating role of a comprehensive lifestyle on the association between exposure to individual and mixed air pollutants and CDs remains unclear. We adopted a more comprehensive and quantitative approach to assessing lifestyle. By constructing a comprehensive lifestyle score, we found that as lifestyle scores increased, the risk of CDs among older adults gradually decreased. We also found that improvements in lifestyle during the follow-up period significantly reduced the risk of CDs in air pollution exposure. At high levels of air pollution exposure, adhering to or transitioning to an intermediate or favourable lifestyle had a more significant protective effect against CDs. Lifestyle factors mitigated the adverse effects of air pollutant exposure on CDs in older adults. In the mediation analysis, lifestyle factors could explain 8.42–9.42% of the association between exposure to single air pollutants and the risk of CDs, and 9.07% of the association for mixed air pollutants and the risk of CDs. Our results suggest that lifestyle mediates the effects of air pollution exposure on CDs. Adopting healthier lifestyles can mitigate air pollution's negative effects on cognitive health. These findings may offer new opportunities to develop effective interventions to reduce the risk of CDs among older adults through comprehensive lifestyle modifications, air pollution prevention, and control strategies.

Notably, social and leisure activities played unique moderating roles in CDs under air pollution exposure. Under high levels of air pollution (Q2–Q5), both social activities (OR = 0.51; 95% CI = 0.31–0.83) and leisure activities (OR = 0.62; 95% CI = 0.44–0.89) were significantly associated with a reduced risk of CDs among the elderly (Table S6 in the **Online Supplementary Document**). Further analysis across different air pollutants showed that, even when exposed to pollutants such as PM_2.5_, SO_4_, O_3_, NO_3_, and NH_4_, social and leisure activities still reduced the risk of CDs. Although the CIs for some data were relatively wide due to factors such as sample size, the overall trend was clear. This indicates that, even in an air-polluted environment, active participation in social activities (*e.g.* square dancing and attending community gatherings) and leisure activities (*e.g.* reading books or newspapers, keeping pets, and gardening chores) positively affected the cognitive health of the elderly. Given that the elderly population generally demonstrates a high level of compliance in accepting and implementing relevant guidance, strengthening publicity and educational efforts to promote social and leisure activities that align with their needs is a highly effective strategy [38]. Older adults should be encouraged to engage more in social activities, such as joining community-organised interest groups and regularly meeting with friends, as well as participating in leisure activities like gardening and moderate housework. These activities not only provide older adults with richer life experiences, but also mitigate the negative impact of air pollution on their cognitive health.

However, several limitations should be considered. First, although the CLHLS is a comprehensive prospective national cohort study, its sample was primarily from China; therefore, the generalisability of the results may be limited. This study was a fixed cohort and did not include newly added participants in subsequent surveys. We also excluded participants who were lost to follow-up or died during the follow-up period, which may lead to better health status and more stable cognitive function for the participants, but there may be survival bias. Second, air pollution exposure was estimated using high-resolution grid models; however, some measurement error may remain [39]. Because of personal privacy concerns, we did not report individual home addresses; the CLHLS questionnaire provided only regional information. Assigning air pollution exposure to participants based on regional averages may result in biased exposure estimates. In addition, lifestyle data relied on self-reporting, which may have introduced recall bias.

Nevertheless, our results have important public health implications. To address the challenges of global ageing and rising prevalence of CDs, we need to develop comprehensive prevention and intervention strategies that consider lifestyle and environmental factors. By improving lifestyle and reducing exposure to air pollution, it may be possible to create healthier living environments for older adults, thereby effectively reducing the risk of CDs. Future studies should further explore the differences in the effects of lifestyle and air pollution on cognitive health across populations, regions, and pollution levels, and how interventions can be optimised through more precise exposure assessments and mechanistic investigations.

## CONCLUSIONS

Using a nationally representative Chinese cohort, we found that lifestyle partially mediated the association between exposure to ambient air pollutants and CDs. Adhering to a healthy lifestyle can significantly reduce the risk of CDs, particularly among individuals with high levels of air pollution. Our results highlight the importance of lifestyle modifications in reducing the burden of CDs, offering an effective strategy to mitigate air pollution's effects on cognitive health. In addition to addressing air pollution at its source, effective policy measures should encourage individuals to adopt healthy lifestyles to protect them from air pollution hazards. Therefore, developing comprehensive air pollution prevention and control strategies and health promotion programmes is crucial to reducing the risk of CDs and protecting the cognitive health of the elderly.

## Additional material


Online Supplementary Document

## Data Availability

**Data availability:** The data sets analysed in this study are publicly available from the CLHLS website: http://opendata.pku.edu.cn/.
