# Supplementary material for: Association between long-term ambient air pollution exposure and cognitive disorders among older adults in China: the mediating role of lifestyle
Source: J Glob Health. 2026 Oct 19;16:04307. doi: 10.7189/jogh.16.04307 (PMC13679344; doi:10.7189/jogh.16.04307)
Supplement: Supplementary file 1 [file jogh-16-04307-s001.pdf]

Supplement to: Zhang Y, Yang J, Zhang Y, Yuan Q, Li Y, Ding R, Song G, Niu Q, Hu Y, Yan Y. Association between long-term ambient air pollution exposure and cognitive disorders among older adults in China: the mediating role of lifestyle. J Glob Health. 2026;16:04307.

Figure S1. Sampling procedure.
Figure S2. Study flowchart.
Figure S3. Directed acyclic graph.
Figure S4. The correlations of different lifestyle factors.
Figure S5. Panel A. The proportion of single ideal factor in different lifestyle groups. Panel B. The proportion of ideal factors in different lifestyle groups.
Figure S6. The mediation effect of lifestyle categories to air pollution in CDs.
Figure S7. Panel A. Positive weighted values of air pollutant exposure for CDs in WQS models. Panel B. Negative weighted values of air pollutant exposure for CDs in WQS models. Panel C. The mediation effect of lifestyle categories to Mix-WQS in CDs. Panel D. Weighted values of air pollutant exposure for CDs in Qgcomp models.
Table S1. The score criteria of different lifestyle factors.
Table S2. The exposure level by quintile of air pollutant.
Table S3. The exposure level of different air pollutants among the study population.
Table S4 The HR (95% Cl) of the associations between lifestyle and cognitive impairment with and without adjustment for ambient air pollutant exposure.
Table S5. Joint effects of lifestyle and air pollutant exposure on the incidence of cognitive impairment.
Table S6. The HRs (95% CIs) of cognitive impairment events associated with each lifestyle factor at different levels of air pollutant exposure.
Table S7. Number and percentage of changes in lifestyle category from baseline to outcome.
Table S8. The HR (95% CI) of the associations between lifestyle dynamic changes and the risk of CDs under exposure to SO_4.
Table S9. The HR (95% CI) of the associations between lifestyle dynamic changes and the risk of CDs under exposure to O_3.
Table S10. The HR (95% CI) of the associations between lifestyle dynamic changes and the risk of CDs under exposure to NO_3.
Table S11. The HR (95% CI) of the associations between lifestyle dynamic changes and the risk of CDs under exposure to NH_4.
Table S12. The association between air pollutants exposure and CDs events in different lifestyle categories
Table S13. Subgroup analysis of the additive interactions analysis of the effect of dichotomized lifestyle on the association between ambient air pollutant exposure and Cognitive impairment in high air pollutant exposure levels (Q2–Q5)
Table S14. Results from WQS and Qgcomp analysis in the air pollutants exposure

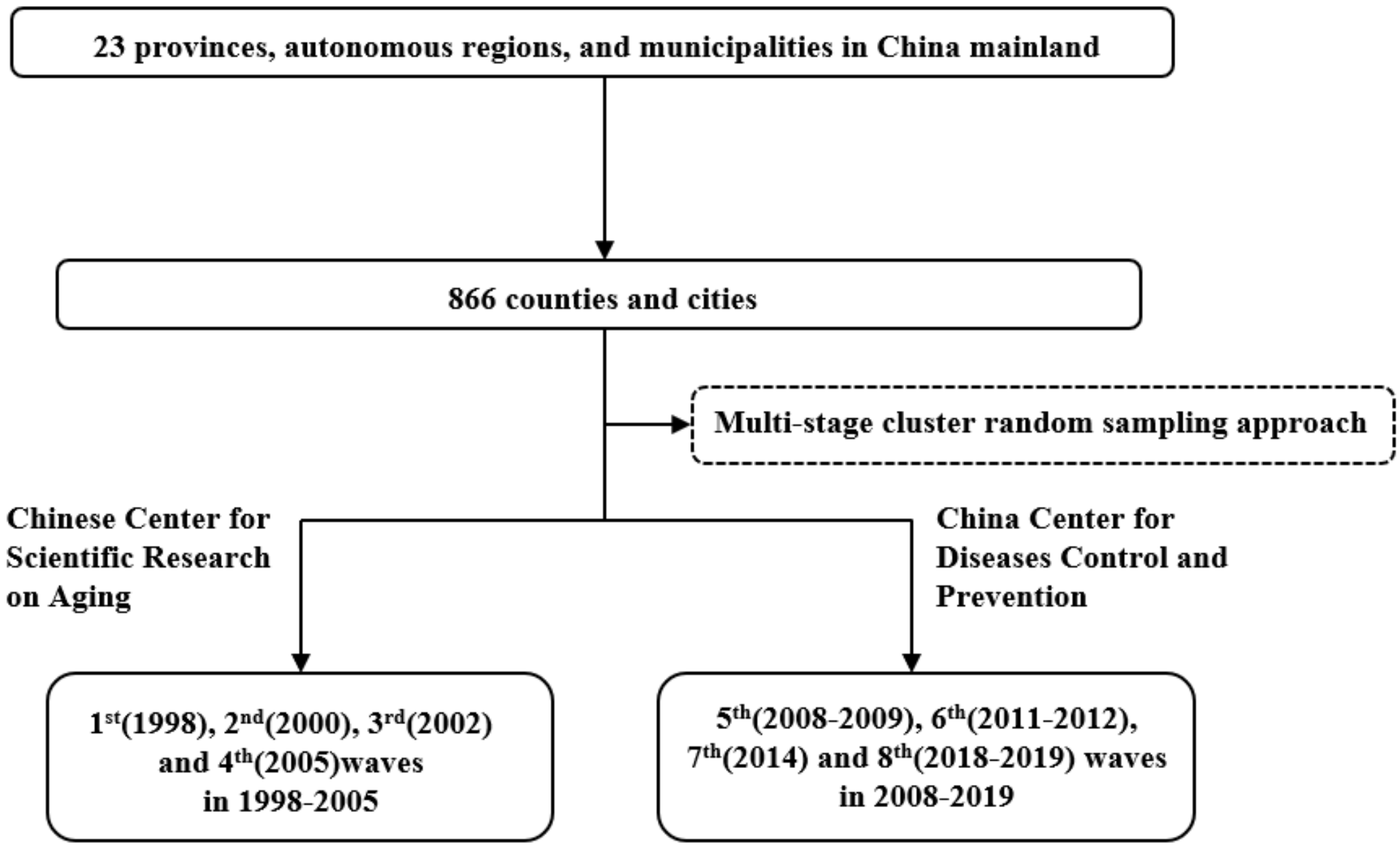

Figure S1. Sampling procedure.

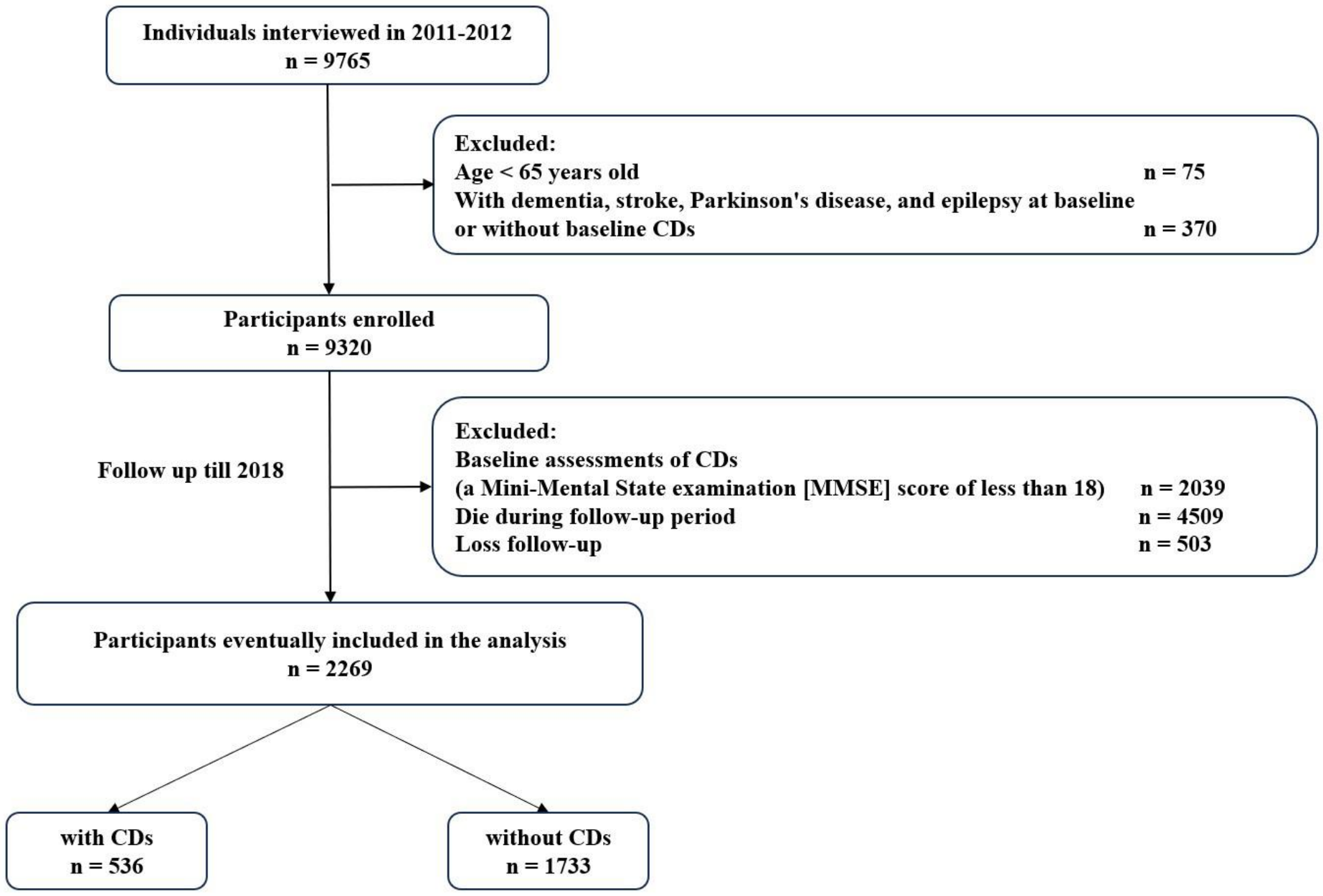

Figure S2. Study flowchart.
CDs Cognitive disorders.

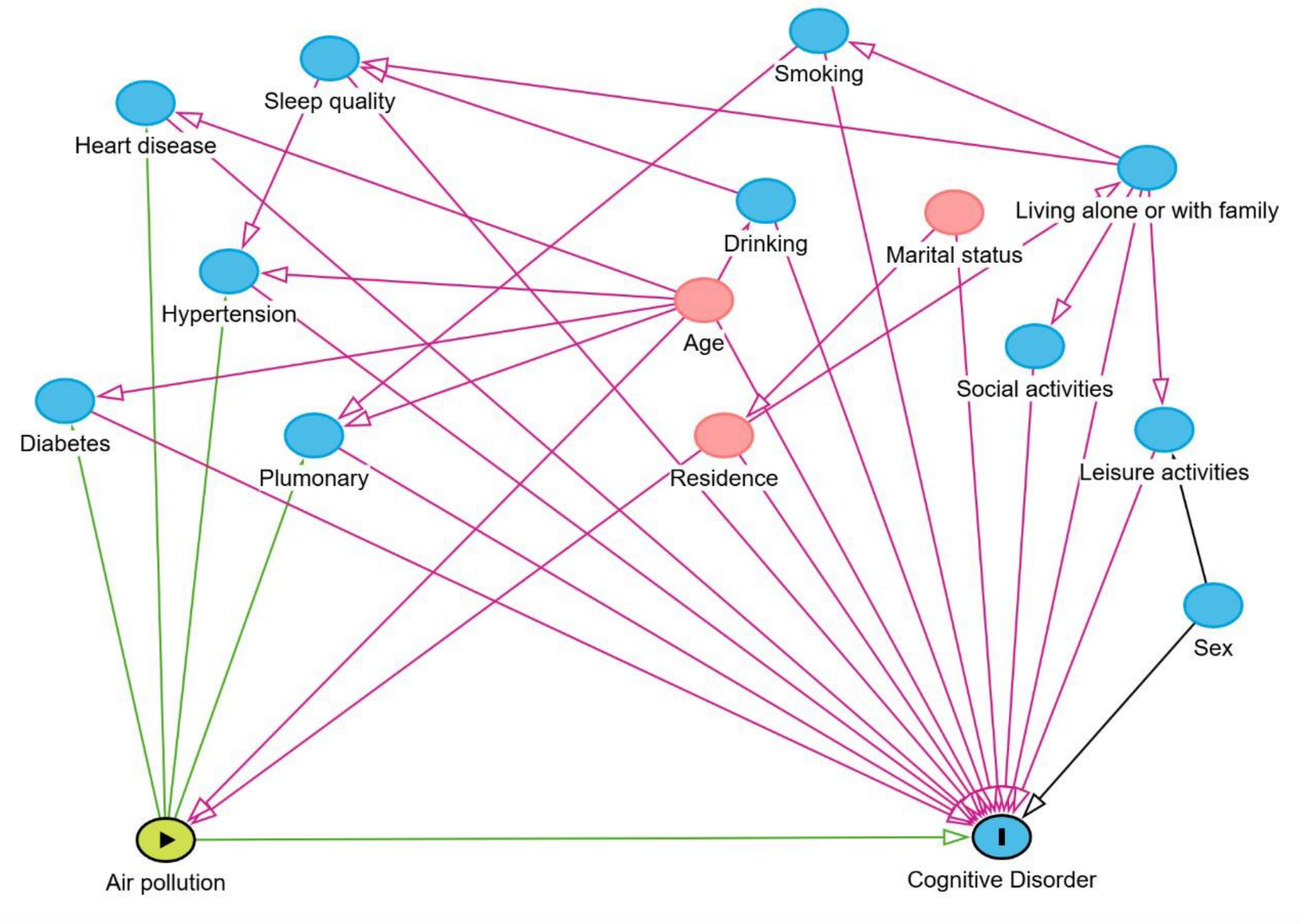

Figure S3. Directed acyclic graph.

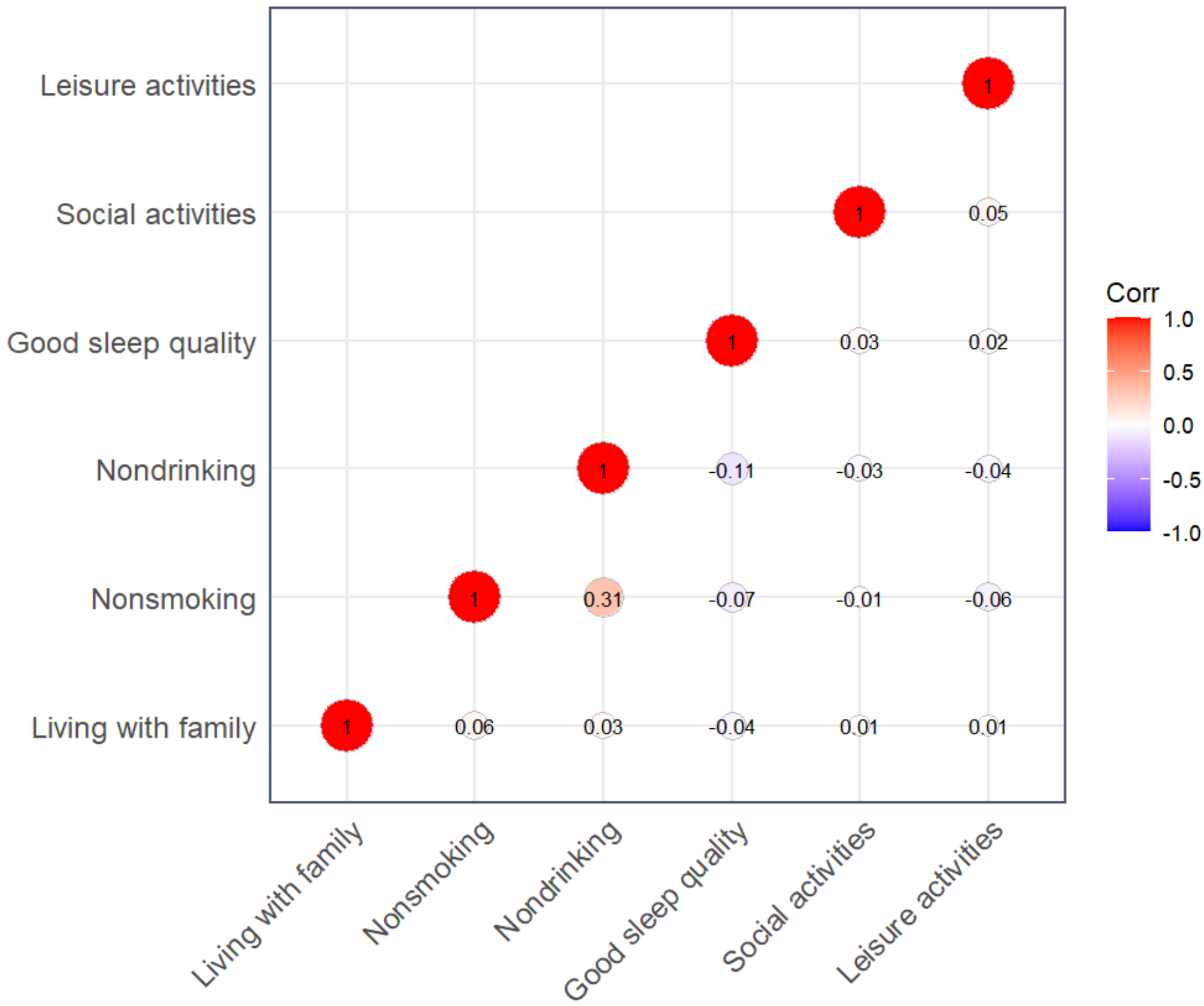

Figure S4. The correlations of different lifestyle factors.

Variables are labelled according to their healthy status (score = 1) as defined in Table S1 (e.g., 'Living with family' corresponds to a favourable 'Residential situation').

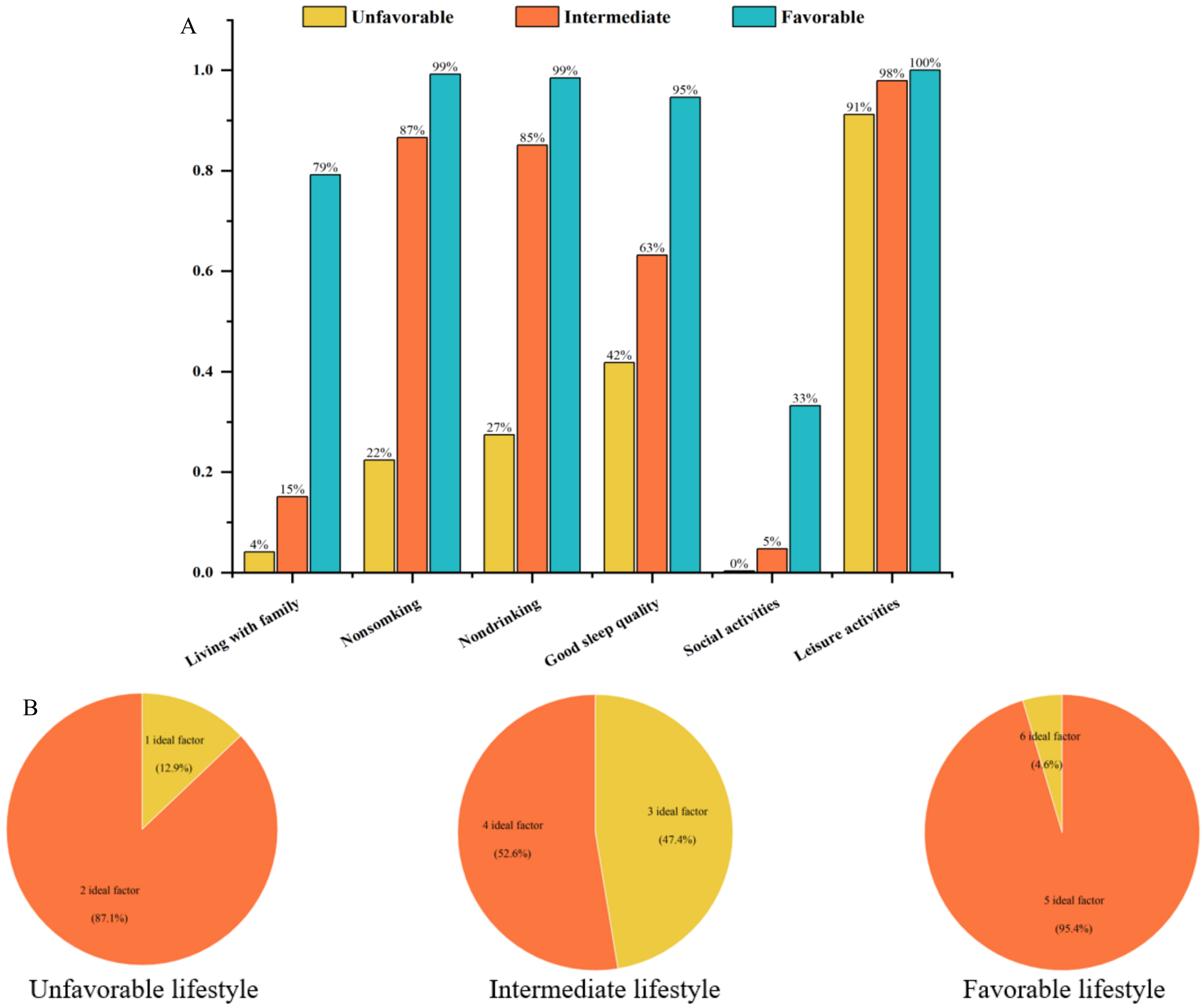

Figure S5. (A) The proportion of single ideal factor in different lifestyle groups. (B) The proportion of ideal factors in different lifestyle groups.

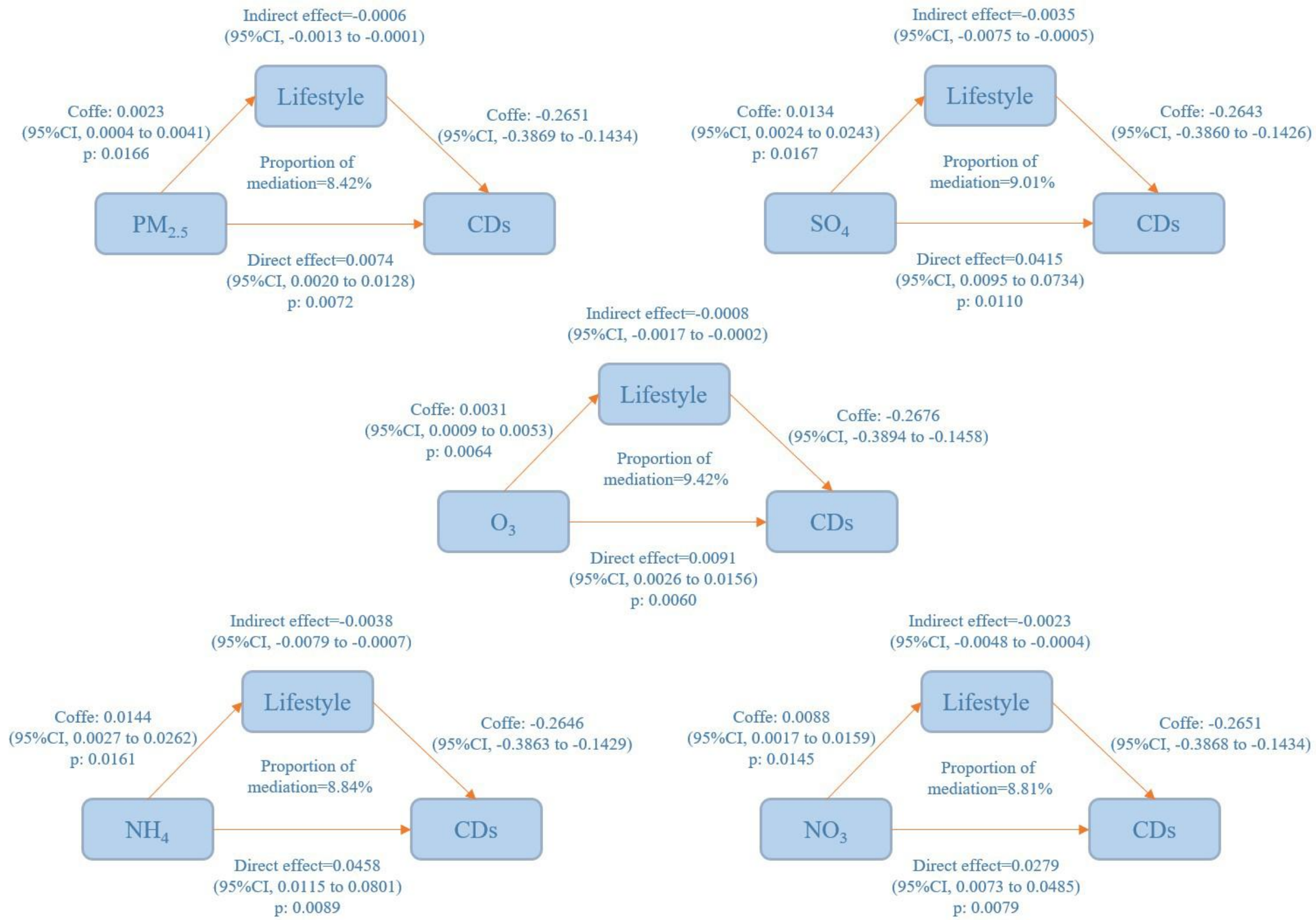

Figure S6. The mediation effect of lifestyle categories to air pollution in CDs.

CDs Cognitive disorders, PM2.5 Particulate matter with an aerodynamic diameter less than 2.5 μm, SO4 Compounds containing sulfate ions, O3 Ozone, NO3 Compounds containing nitrate ions, NH4 Compounds containing ammonium ions.

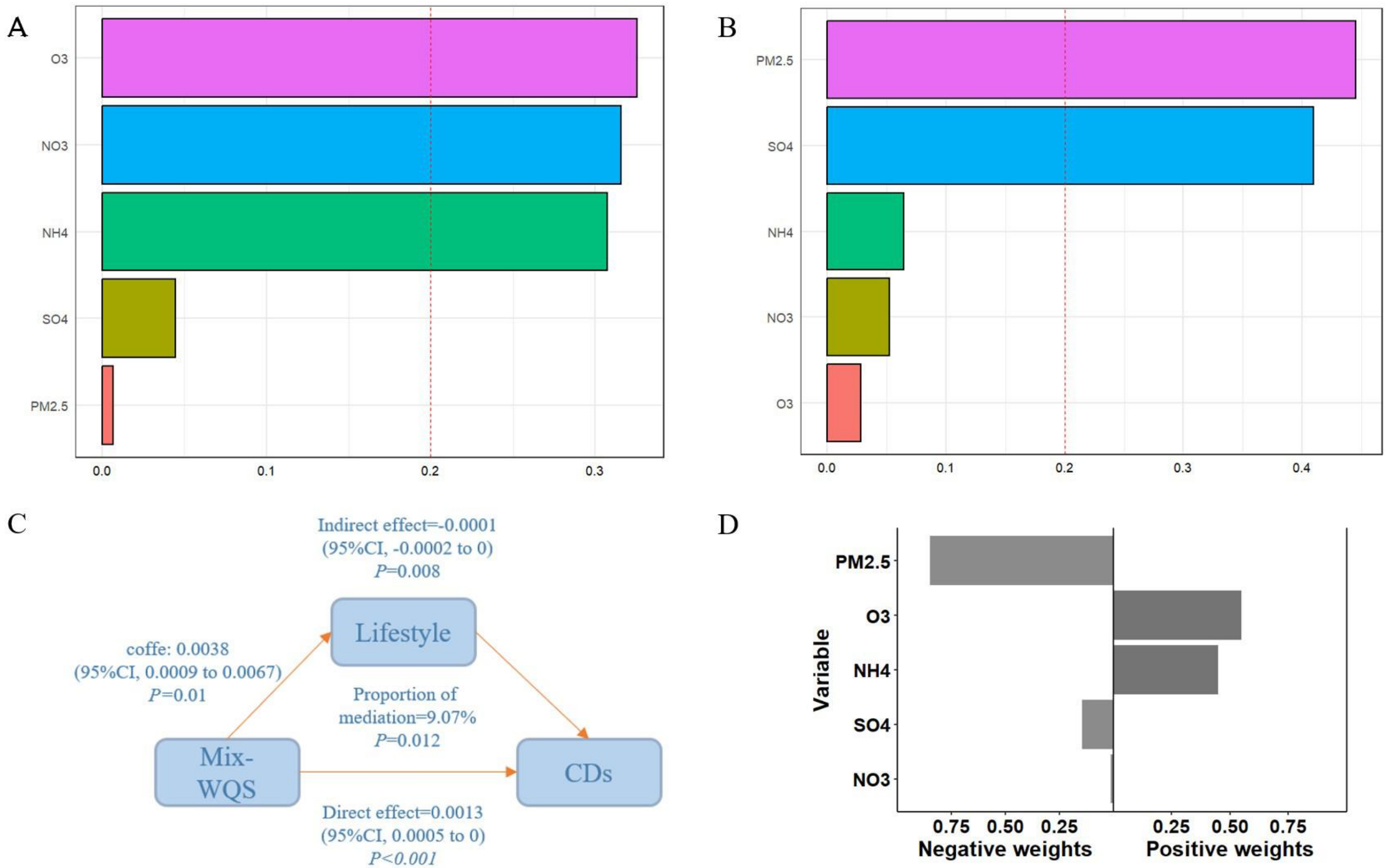

Figure S7. (A) Positive weighted values of air pollutant exposure for CDs in WQS models. (B) Negative weighted values of air pollutant exposure for CDs in WQS models. (C) The mediation effect of lifestyle categories to Mix-WQS in CDs. (D) Weighted values of air pollutant exposure for CDs in Qgcomp models. All Models were adjusted for age, sex, marital status, residence, hypertension, diabetes, heart disease, and chronic lung disease. $PM_{2.5}$ Particulate matter with an aerodynamic diameter less than 2.5 μm, SO_4 Compounds containing sulfate ions, O_3 Ozone, NO_3 Compounds containing nitrate ions, NH_4 Compounds containing ammonium ions. Mix-WQS The sum of the products of the weights obtained from the WQS models and concentrations of various air pollutants.

Table S1. The score criteria of different lifestyle factors

Variables	Score	
	0	1
Smoking	Yes	None
Drinking	Yes	None
Residential situation	Living alone	Living with family (with household member (s) or in an institution)
Sleep quality	Bad sleep quality (so so, bad or very bad)	Good sleep quality (very good or good)
Social activities	None, sometimes or at least once a month	almost everyday or once for a week
Leisure activities	None, sometimes or at least once a month	Leisure activity (including do housework, grow vegetables, garden housework, read books or newspapers, keep pets, play cards, watch TV) almost everyday or once for a week

Table S2. The exposure level by quintile of air pollutant

Air pollutants	Minimum	Maximum	Mean(SD)	Median(IQR)
$PM_{2.5}$, μg/m^3				
Q1(n = 428)	18.23	31.22	27.48(5.46)	31.00(4.59)
Q2(n = 337)	31.74	31.74	31.74(0.00)	31.74(0.00)
Q3(n = 569)	35.85	56.67	45.78(8.00)	47.48(16.06)
Q4(n = 322)	58.15	65.67	63.89(2.48)	64.98(0.00)
Q5(n = 613)	73.31	86.83	77.32(3.26)	79.48(6.17)
Q2-Q5(n = 1841)	31.74	86.83	56.88(18.05)	58.15(37.46)
SO_4, μg/m^3				
Q1(n = 339)	4.08	6.19	5.44(0.96)	6.19(2.11)
Q2(n = 439)	6.56	6.65	6.58(0.04)	6.56(0.00)
Q3(n = 507)	7.4	11.11	8.98(1.44)	9.66(2.87)
Q4(n = 383)	11.15	13.37	11.72(0.48)	11.96(0.81)
Q5(n = 601)	13.71	14.53	14.21(0.40)	14.53(0.82)
Q2-Q5(n = 1930)	6.56	14.53	10.61(3.05)	11.15(6.30)
O_3, μg/m^3				
Q1(n = 312)	94.05	100.65	99.10(2.04)	100.65(3.02)
Q2(n = 475)	102.53	111.35	104.24(3.14)	102.53(0.38)
Q3(n = 546)	112.15	123.92	117.46(4.03)	119.82(6.77)
Q4(n = 279)	126.36	134.51	133.11(2.99)	134.51(0.00)
Q5(n = 657)	135.29	146.96	141.22(4.40)	144.28(8.98)
Q2-Q5(n = 1957)	102.53	146.96	124.46(15.25)	123.30(23.14)
NO_3, μg/m^3				
Q1(n = 222)	2.61	5.81	3.93(1.30)	4.52(2.73)
Q2(n = 543)	5.81	6.32	5.99(0.24)	5.81(0.51)
Q3(n = 493)	7.31	11.48	9.27(1.60)	10.44(3.32)

Q4(n = 410)	12.8	15.63	14.36(1.19)	15.32(2.53)
Q5(n = 601)	17.37	18.41	18.02(0.51)	18.41(1.04)
Q2-Q5(n = 2047)	5..33	18.41	11.99(4.91)	11.48(11.05)
NH_4, μg/m^3				
Q1(n = 584)	2.34	4.82	4.23(4.82)	4.82(0.71)
Q2(n = 181)	4.91	4.91	4.91(0.00)	4.91(0.00)
Q3(n = 534)	5.49	8.88	6.94(1.21)	7.60(2.54)
Q4(n = 369)	9.04	10.32	9.81(0.54)	10.20(1.16)
Q5(n = 601)	11.64	12.14	11.95(0.25)	12.14(0.51)
Q2-Q5(n = 1685)	4.91	12.14	9.14(2.63)	10.03(5.28)

IQR Interquartile range, $PM_{2.5}$ Particulate matter with an aerodynamic diameter less than 2.5 μm, SO_4 Compounds containing sulfate ions, O_3 Ozone, NO_3 Compounds containing nitrate ions, NH_4 Compounds containing ammonium ions.

Table S3. The exposure level of different air pollutants among the study population

Air pollutants	Minimum	10%	25%	50%	75%	90%	Maximum	Mean (SD)	Median (IQR)
Total(N = 2269)									
$PM_{2.5}$, μg/m^3	18.23	31.00	31.74	51.91	73.31	79.48	86.83	51.33(20.06)	51.91(41.57)
SO_4, μg/m^3	4.08	6.19	6.56	10.27	13.71	14.53	14.53	9.84(3.38)	10.27(7.14)
O_3, μg/m^3	94.05	100.65	102.53	119.82	135.29	144.28	146.96	120.97(16.66)	119.82(32.761)
NO_3, μg/m^3	2.61	5.81	5.90	10.63	17.37	18.41	18.41	11.20(5.26)	10.63(11.47)
NH_4, μg/m^3	2.34	4.42	4.82	7.75	11.64	12.14	12.14	7.88(3.16)	7.75(6.82)
Unfavourable lifestyle (N = 340)									
$PM_{2.5}$, μg/m^3	18.23	31.00	31.74	51.91	73.31	79.48	79.48	51.99(20.83)	51.91(41.54)
SO_4, μg/m^3	4.08	6.19	6.56	10.83	13.71	14.53	14.53	9.95(3.53)	10.83(7.14)
O_3, μg/m^3	94.05	100.65	102.53	119.95	135.29	144.28	146.58	121.54(17.49)	119.95(32.76)
NO_3, μg/m^3	2.61	5.34	5.81	10.63	17.37	18.41	18.41	11.39(5.49)	10.63(11.56)
NH_4, μg/m^3	2.34	4.42	4.82	7.75	11.64	12.14	12.14	7.98(3.29)	7.75(6.82)
Intermediate lifestyle (N = 1670)									
$PM_{2.5}$, μg/m^3	18.23	30.99	31.74	47.48	73.31	79.48	86.83	51.08(19.95)	47.48(41.75)

SO_4, μg/m^3	4.08	6.19	6.56	9.66	13.71	14.53	14.53	9.80(3.37)	9.66(7.14)
O_3, μg/m^3	94.05	100.65	102.53	119.82	135.29	144.28	146.96	120.70(16.53)	119.82(32.76)
NO_3, μg/m^3	2.61	5.81	5.81	10.44	17.37	18.41	18.41	11.13(5.23)	10.44(11.56)
NH_4, μg/m^3	2.34	4.42	4.82	7.60	11.64	12.14	12.14	7.84(3.14)	7.60(6.82)
Favourable lifestyle (N = 259)									
$PM_{2.5}$, μg/m^3	18.23	31.00	31.74	51.91	73.31	79.48	86.83	52.07(19.80)	51.91(41.57)
SO_4, μg/m^3	4.08	6.19	6.56	10.27	13.71	14.53	14.53	9.96(3.32)	10.27(7.14)
O_3, μg/m^3	94.05	100.65	102.92	119.95	135.29	144.28	146.96	121.95(5.15)	119.95(32.38)
NO_3, μg/m^3	2.61	5.81	6.32	10.63	17.37	18.41	18.41	11.38(5.15)	10.63(11.05)
NH_4, μg/m^3	2.34	4.42	4.91	8.02	11.64	12.14	12.14	7.98(3.09)	8.03(6.73)

IQR Interquartile range, $PM_{2.5}$ Particulate matter with an aerodynamic diameter less than 2.5 μm, SO_4 Compounds containing sulfate ions, O_3 Ozone, NO_3 Compounds containing nitrate ions, NH_4 Compounds containing ammonium ions.

Table S4 The HR (95% Cl) of the associations between lifestyle and cognitive impairment with and without adjustment for ambient air pollutant exposure

Lifestyle	Unadjusted for air pollutants	Adjusted for air pollutants [a]
Unfavourable	Reference	Reference
Intermediate	0.958(0.753-1.219)	0.732(0.569-0.940)
Favourable	0.784(0.549-1.122)	0.488(0.336-0.710)

CI Confidence interval, CDs Cognitive disorders, HR Hazard ratio.

[a] Model adjusted for age, sex, marital status, residence, hypertension, diabetes, heart disease, chronic lung disease, and air pollutants ($PM_{2.5}$, SO_4, O_3, NO_3 and NH_4).

Table S5. Joint effects of lifestyle and air pollutant exposure on the incidence of cognitive impairment

Groups	Lifestyle	$PM_{2.5}$	SO_4	O_3	NO_3	NH_4
	Favourable	0.49(0.21-1.11)	0.55(0.20-1.49)	0.66(0.23-1.89)	0.39(0.12-1.25)	0.33(0.13-0.81)
Q1	Intermediate	0.86(0.49-1.50)	0.94(0.49-1.93)	1.14(0.56-2.34)	0.63(0.30-1.31)	0.61(0.37-1.02)
	Unfavourable	Reference	Reference	Reference	Reference	Reference
	Favourable	0.11(0.01-0.85)	0.20(0.06-0.61)	0.28(0.10-0.81)	0.40(0.16-0.98)	0.80(0.24-2.65)
Q2	Intermediate	0.58(0.28-1.94)	0.55(0.31-0.98)	0.58(0.32-1.06)	0.84(0.49-1.43)	1.28(0.54-3.01)
	Unfavourable	Reference	Reference	Reference	Reference	Reference
	Favourable	0.55(0.26-1.16)	0.61(0.30-1.25)	0.53(0.27-1.07)	0.52(0.24-1.12)	0.61(0.30-1.22)
Q3	Intermediate	0.76(0.45-1.29)	0.56(0.33-0.96)	0.60(0.37-0.99)	0.58(0.33-1.02)	0.54(0.32-0.91)
	Unfavourable	Reference	Reference	Reference	Reference	Reference
	Favourable	1.23(0.54-2.80)	1.23(0.53-2.83)	1.27(0.46-3.54)	1.28(0.60-2.73)	1.21(0.51-2.91)
Q4	Intermediate	0.74(0.37-1.45)	1.12(0.58-2.13)	1.30(0.57-3.00)	0.98(0.54-1.79)	1.20(0.61-2.35)
	Unfavourable	Reference	Reference	Reference	Reference	Reference
	Favourable	0.24(0.11-0.52)	0.22(0.10-0.49)	0.30(0.15-0.61)	0.22(0.10-0.49)	0.22(0.10-0.48)
Q5	Intermediate	0.62(0.39-0.99)	0.62(0.39-0.99)	0.63(0.41-0.98)	0.62(0.39-0.99)	0.62(0.39-0.99)
	Unfavourable	Reference	Reference	Reference	Reference	Reference

	Favourable [a]	0.47(0.31-0.72)	0.47(0.32-0.71)	0.46(0.31-0.69)	0.49(0.33-0.72)	0.52(0.34-0.78)
Q2-Q5	Intermediate [a]	0.70(0.53-0.92)	0.69(0.53-0.90)	0.68(0.52-0.89)	0.74(0.56-0.96)	0.75(0.57-1.01)
	Unfavourable [b]	Reference	Reference	Reference	Reference	Reference

Data presented as HR (95% CI).

CI Confidence interval, CDs Cognitive disorders, HR Hazard ratio, $PM_{2.5}$ Particulate matter with an aerodynamic diameter less than 2.5 μm, SO_4 Compounds containing sulfate ions, O_3 Ozone, NO_3 Compounds containing nitrate ions, NH_4 Compounds containing ammonium ions.

[a] HR Reduction Rate = (1 - HR) * 100%

[b] The HR for the reference group is assumed to be 1.

Table S6. The HRs (95% CIs) of cognitive impairment events associated with each lifestyle factor at different levels of air pollutant exposure

Lifestyle	All	$PM_{2.5}$		SO_4		O_3		NO_3		NH_4	
		Q1	Q2-Q5	Q1	Q2-Q5	Q1	Q2-Q5	Q1	Q2-Q5	Q1	Q2-Q5
Non smoking	0.79 (0.61-1.02)	0.74 (0.44-1.22)	0.84 (0.62-1.12)	0.89 (0.47-1.70)	0.78 (0.59-1.02)	0.96 (0.49-1.89)	0.77 (0.59-1.02)	0.44 (0.22-0.90)	0.87 (0.66-1.14)	0.49 (0.29-0.83)	0.91 (0.68-1.21)
Non drinking	0.89 (0.70-1.11)	1.04 (0.59-1.82)	0.85 (0.66-1.09)	1.36 (0.66-2.83)	0.84 (0.66-1.08)	1.40 (0.65-3.01)	0.84 (0.66-1.07)	0.76 (0.34-1.78)	0.89 (0.70-1.13)	0.87 (0.51-1.49)	0.87 (0.68-1.13)
Living with family	0.78 (0.61-0.98)	0.64 (0.39-1.03)	0.80 (0.61-1.04)	0.67 (0.39-1.16)	0.79 (0.61-1.02)	0.64 (0.36-1.13)	0.79 (0.61-1.02)	0.61 (0.32-1.20)	0.78 (0.61-0.99)	0.73 (0.45-1.19)	0.78 (0.60-1.02)
Good sleep quality	0.91 (0.76-1.10)	1.01 (0.68-1.50)	0.89 (0.72-0.10)	0.98 (0.62-1.55)	0.91 (0.74-1.12)	1.07 (0.67-1.70)	0.90 (0.73-1.10)	0.88 (0.50-1.54)	0.91 (0.75-1.11)	0.98 (0.67-1.43)	0.90 (0.73-1.11)
Social activities	0.51 (0.31-0.83)	0.45 (0.18-1.13)	0.51 (0.29-0.91)	0.28 (0.07-1.17)	0.56 (0.34-0.94)	0.46 (0.11-1.90)	0.51 (0.31-0.86)	0.45 (0.13-1.57)	0.50 (0.29-0.85)	0.53 (0.19-1.49)	0.49 (0.28-0.86)
Leisure activities	0.62 (0.44-0.89)	0.80 (0.31-2.05)	0.60 (0.40-0.88)	0.93 (0.32-2.69)	0.59 (0.40-0.87)	1.20 (0.36-3.95)	0.58 (0.40-0.85)	0.71 (0.21-2.45)	0.63 (0.43-0.91)	0.70 (0.37-1.42)	0.61 (0.40-0.93)
Get 1 point in lifestyle score	Reference	Reference	Reference	Reference	Reference	Reference	Reference	Reference	Reference	Reference	Reference
Get 2 point in	0.76 (0.39-1.48)	1.11 (0.25-4.92)	0.69 (0.33-1.45)	0.72 (0.15-3.41)	0.74 (0.35-1.56)	1.44 (0.18-11.56)	0.67 (0.33-1.36)	0.56 (0.63-4.92)	0.77 (0.38-1.55)	2.45 (0.32-18.56)	0.62 (0.30-1.27)

lifestyle score											
Get 3 point in lifestyle score	0.60 (0.31-1.14)	1.04 (0.24-4.47)	0.52 (0.25-1.07)	0.75 (0.17-3.37)	0.56 (0.27-1.14)	1.58 (0.20-12.23)	0.51 (0.26-0.99)	0.52 (0.06-4.34)	0.59 (0.30-1.17)	1.52 (0.21-11.21)	0.52 (0.26-1.03)
Get 4 point in lifestyle score	0.54 (0.28-1.03)	0.87 (0.20-3.75)	0.48 (0.24-0.99)	0.73 (0.16-3.25)	0.50 (0.24-1.03)	1.59 (0.21-12.28)	0.45 (0.23-0.89)	0.25 (0.03-2.16)	0.57 (0.29-1.13)	1.29 (0.17-9.63)	0.48 (0.24-0.95)
Get 5 point in lifestyle score	0.39 (0.92-0.78)	0.57 (0.12-2.72)	0.34 (0.16-0.75)	0.43 (0.08-2.22)	0.37 (0.17-0.80)	0.94 (0.11-8.3)	0.33 (0.16-0.69)	0.26 (0.03-2.54)	0.39 (0.19-0.82)	0.78 (0.09-6.60)	0.35 (0.17-0.73)
Get 6 point in lifestyle score	0.17 (0.02-1.37)	Non[a]	0.19 (0.02-1.55)	Non	0.18 (0.023-1.47)	Non	0.16 (0.02-1.25)	Non	0.20 (0.03-1.61)	Non	0.17 (0.02-1.31)

CI Confidence interval, CDs Cognitive disorders, HR Hazard ratio, $PM_{2.5}$ Particulate matter with an aerodynamic diameter less than 2.5 μm, SO_4 Compounds containing sulfate ions, O_3 Ozone, NO_3 Compounds containing nitrate ions, NH_4 Compounds containing ammonium ions.

[a] The sample size for a lifestyle score of 6 is relatively small, especially in areas with low air pollution exposure. May affect the accuracy and reliability of the results, and the results are only used to determine trends.

Table S7. Number and percentage of changes in lifestyle category from baseline to outcome

Baseline	Outcome	N(%)
Unfavourable lifestyle	Unfavourable lifestyle	73(3.21)
	Intermediate lifestyle	230(10.14)
	Favourable lifestyle	37(1.63)
Intermediate lifestyle	Unfavourable lifestyle	139(6.13)
	Intermediate lifestyle	1106(48.74)
	Favourable lifestyle	425(18.73)
Favourable lifestyle	Unfavourable lifestyle	26(1.15)
	Intermediate lifestyle	169(7.45)
	Favourable lifestyle	64(2.82)

Table S8. The HR (95% CI) of the associations between lifestyle dynamic changes and the risk of CDs under exposure to SO_4

Lifestyle category in baseline	Lifestyle category in outcome	events/n	Q1		Q2-Q5	
			HR(95%CI)	*P*	HR(95%CI)	*P*
Unfavourable lifestyle	Unfavourable lifestyle	19/73	1(reference)		1(reference)	
	Intermediate lifestyle	53/230	0.95(0.22-3.99)	0.945	1.07(0.59-1.93)	0.822
	Favourable lifestyle	8/37	2.26(0.36-14.13)	0.382	0.98(0.34-2.76)	0.962
Intermediate lifestyle	Unfavourable lifestyle	67/139	1(reference)		1(reference)	
	Intermediate lifestyle	252/1106	0.43(0.19-0.94)	0.036	0.40(0.30-0.53)	<0.001
	Favourable lifestyle	60/425	0.30(0.11-0.80)	0.017	0.29(0.20-0.42)	<0.001
Favourable lifestyle	Unfavourable lifestyle	5/26	1(reference)		1(reference)	
	Intermediate lifestyle	36/169	0.16(0.01-20.26)	0.461	1.22(0.42-3.57)	0.714
	Favourable lifestyle	7/64	-	0.975	0.99(0.28-3.60)	0.994

HR and *P* were adjusted for age, sex, marital status, residence, hypertension, diabetes, heart disease, chronic lung disease.

- The sample size for this category is too small or the outcome is consistent

Table S9. The HR (95% CI) of the associations between lifestyle dynamic changes and the risk of CDs under exposure to O_3

Lifestyle category in baseline	Lifestyle category in outcome	events/n	Q1		Q2-Q5	
			HR(95%CI)	*P*	HR(95%CI)	*P*
Unfavourable lifestyle	Unfavourable lifestyle	19/73	1(reference)		1(reference)	
	Intermediate lifestyle	53/230	0.77(0.16-3.73)	0.749	1.10(0.61-1.98)	0.759
	Favourable lifestyle	8/37	2.38(0.36-15.87)	0.372	0.98(0.35-2.78)	0.97
Intermediate lifestyle	Unfavourable lifestyle	67/139	1(reference)		1(reference)	
	Intermediate lifestyle	252/1106	0.46(0.21-1.01)	0.052	0.40(0.30-0.54)	<0.001
	Favourable lifestyle	60/425	0.33(0.12-0.89)	0.029	0.29(0.20-0.42)	<0.001
Favourable lifestyle	Unfavourable lifestyle	5/26	1(reference)		1(reference)	
	Intermediate lifestyle	36/169	1.27(0.002-876.38)	0.942	1.27(0.43-3.70)	0.666
	Favourable lifestyle	7/64	-	0.983	1.03(0.28-3.70)	0.969

HR and *P* were adjusted for age, sex, marital status, residence, hypertension, diabetes, heart disease, chronic lung disease.

- The sample size for this category is too small or the outcome is consistent

Table S10. The HR (95% CI) of the associations between lifestyle dynamic changes and the risk of CDs under exposure to NO_3

Lifestyle category in baseline	Lifestyle category in outcome	events/n	Q1		Q2-Q5	
			HR(95%CI)	*P*	HR(95%CI)	*P*
Unfavourable lifestyle	Unfavourable lifestyle	19/73	1(reference)		1(reference)	
	Intermediate lifestyle	53/230	1.98(0.34-11.50)	0.453	1.02(0.58-1.81)	0.940
	Favourable lifestyle	8/37	-	0.968	0.97(0.39-2.38)	0.942
Intermediate lifestyle	Unfavourable lifestyle	67/139	1(reference)		1(reference)	
	Intermediate lifestyle	252/1106	0.39(0.12-1.24)	0.110	0.40(0.30-0.54)	<0.001
	Favourable lifestyle	60/425	0.59(0.16-2.17)	0.431	0.26(0.18-0.38)	<0.001
Favourable lifestyle	Unfavourable lifestyle	5/26	1(reference)		1(reference)	
	Intermediate lifestyle	36/169	-	0.148	1.09(0.38-3.16)	0.876
	Favourable lifestyle	7/64	-	0.974	0.82(0.23-2.97)	0.767

HR and *P* were adjusted for age, sex, marital status, residence, hypertension, diabetes, heart disease, chronic lung disease.
- The sample size for this category is too small or the outcome is consistent

Table S11. The HR (95% CI) of the associations between lifestyle dynamic changes and the risk of CDs under exposure to NH_4

Lifestyle category in baseline	Lifestyle category in outcome	events/n	Q1		Q2-Q5	
			HR(95%CI)	*P*	HR(95%CI)	*P*
Unfavourable lifestyle	Unfavourable lifestyle	19/73	1(reference)		1(reference)	
	Intermediate lifestyle	53/230	1.80(0.36-8.99)	0.475	1.01(0.57-1.79)	0.969
	Favourable lifestyle	8/37	-	0.957	0.95(0.38-2.33)	0.903
Intermediate lifestyle	Unfavourable lifestyle	67/139	1(reference)		1(reference)	
	Intermediate lifestyle	252/1106	0.30(0.11-0.84)	0.022	0.41(0.30-0.54)	<0.001
	Favourable lifestyle	60/425	0.36(0.11-1.21)	0.098	0.27(0.18-0.39)	<0.001
Favourable lifestyle	Unfavourable lifestyle	5/26	1(reference)		1(reference)	
	Intermediate lifestyle	36/169	0.55(0.06-4.83)	0.589	1.40(0.41-4.76)	0.588
	Favourable lifestyle	7/64	-	0.999	1.03(0.25-4.26)	0.964

HR and *P* were adjusted for age, sex, marital status, residence, hypertension, diabetes, heart disease, chronic lung disease.

- The sample size for this category is too small or the outcome is consistent

Table S12. The association between air pollutants exposure and CDs events in different lifestyle categories

Air pollutants	Unfavourable HR (95% CI)	Intermediate HR (95% CI)	Favourable HR (95% CI)
$PM_{2.5}$			
Model 1	1.001(0.991-1.012)	1.001(0.996-1.006)	1.004(0.990-1.019)
P value	0.805	0.662	0.553
Model 2	1.008(0.997-1.020)	1.005(1.001-1.011)	1.006(0.992-1.021)
P value	<0.001	<0.001	0.003
SO_4			
Model 1	1.010(0.949-1.075)	1.006(0.976-1.036)	1.012(0.929-1.101)
P value	0.746	0.719	0.789
Model 2	1.049(0.982-1.122)	1.033(1.002-1.064)	1.021(0.937-1.112)
P value	<0.001	<0.001	0.003
O_3			
Model 1	1.003(0.991-1.016)	1.002(0.996-1.016)	1.006(0.989-1.024)
P value	0.611	0.581	0.466
Model 2	1.012(0.998-1.025)	1.006(1.001-1.012)	1.009(0.992-1.027)
P value	<0.001	<0.001	0.002
NO_3			
Model 1	1.002(0.963-1.043)	1.006(0.986-1.025)	1.008(0.954-1.065)
P value	0.926	0.571	0.779
Model 2	1.028(0.985-1.073)	1.023(1.003-1.042)	1.014(0.960-1.071)
P value	<0.001	<0.001	0.003
NH_4			
Model 1	1.005(0.940-1.075)	1.008(0.976-1.041)	1.010(0.922-1.107)
P value	0.88	0.63	0.827
Model 2	1.048(0.975-1.126)	1.037(1.005-1.071)	1.020(0.931-1.118)
P value	<0.001	<0.001	0.003

Model 1: crude model

Model 2: adjusted for age, sex, marital status, residence, hypertension, diabetes, heart disease, and chronic lung disease

CI Confidence interval, CDs Cognitive disorders, $PM_{2.5}$ Particulate matter with an aerodynamic diameter less than 2.5 μm, SO_4 Compounds containing sulfate ions, O_3 Ozone, NO_3 Compounds containing nitrate ions, NH_4 Compounds containing ammonium ions.

Table S13. Subgroup analysis of the additive interactions analysis of the effect of dichotomized lifestyle on the association between ambient air pollutant exposure and Cognitive impairment in high air pollutant exposure levels (Q2–Q5)

Subgroup	n	HR (95% CI) [a]	*P* for interaction
$PM_{2.5}$			0.276
Age			
< 75 years old	901	1.06(0.60-1.86)	
⩾75 years old	940	0.80(0.59-1.09)	
Sex			0.008
Male	903	0.80(0.56-1.16)	
Female	938	0.53(0.35-0.81)	
SO_4			
Age			0.203
< 75 years old	933	0.91(0.54-1.54)	
⩾ 75 years old	997	0.82(0.61-1.1)	
Sex			0.005
Male	949	0.84(0.53-1.11)	
Female	981	0.54(0.36-0.82)	
O_3			
Age			0.166
< 75 years old	943	0.87(0.52-1.45)	
⩾ 75 years old	1014	0.82(0.61-1.11)	
Sex			0.003
Male	964	0.54(0.36-0.82)	
Female	993	0.76(0.54-1.08)	
NO_3			
Age			0.453
< 75 years old	980	1.08(0.62-1.86)	
⩾ 75 years old	1067	0.86(0.65-1.15)	
Sex			0.012
Male	1000	0.82(0.58-1.17)	
Female	1047	0.56(0.38-0.84)	
NH_4			
Age			0.5
< 75 years old	805	0.96(0.55-1.66)	
⩾ 75 years old	880	0.89(0.64-1.22)	
Sex			0.048
Male	827	0.85(0.59-1.24)	
Female	858	0.60(0.38-0.93)	

[a] HR (95% CI) comparing those with intermediate or favourable lifestyle versus those with unfavourable lifestyle in high levels of air pollution (Q2–Q5) were shown.

CI Confidence interval, CDs Cognitive disorders, HR Hazard ratio, $PM_{2.5}$ Particulate matter with an aerodynamic diameter less than 2.5 μm, SO_4 Compounds containing sulfate ions, O_3 Ozone, NO_3 Compounds containing nitrate ions, NH_4 Compounds containing ammonium ions

Table S14. Results from WQS and Qgcomp analysis in the air pollutants exposure

Outcome	OR	95% CI	*P* value
WQS			
Positive model	1.151	(1.008-1.314)	0.037
Negative model	1.129	(0.994-1.283)	0.061
Qgcomp			
Model	1.219	(1.089-1.365)	<0.001

OR odds ratio, CI confidence interval, OR estimates represent the odds ratios of CDs when the WQS index was increased by one quartile.
